# Translational Advances in Oncogene and Tumor-Suppressor Gene Research

**DOI:** 10.3390/cancers17061008

**Published:** 2025-03-17

**Authors:** Radoslav Stojchevski, Edward Agus Sutanto, Rinni Sutanto, Nikola Hadzi-Petrushev, Mitko Mladenov, Sajal Raj Singh, Jitendra Kumar Sinha, Shampa Ghosh, Bhuvaneshwar Yarlagadda, Krishna Kumar Singh, Prashant Verma, Sonali Sengupta, Rakesh Bhaskar, Dimiter Avtanski

**Affiliations:** 1 Friedman Diabetes Institute, Lenox Hill Hospital, Northwell Health, New York, NY 10022, USA; rstojchevski@northwell.edu; 2 Feinstein Institutes for Medical Research, Manhasset, NY 11030, USA; 3 Donald and Barbara Zucker School of Medicine at Hofstra/Northwell, Hempstead, NY 11549, USA; 4 CUNY School of Medicine, The City College of New York, 160 Convent Avenue, New York, NY 10031, USA; esutant000@citymail.cuny.edu; 5 New York Institute of Technology College of Osteopathic Medicine, Glen Head, NY 11545, USA; rsutanto@nyit.edu; 6 Faculty of Natural Sciences and Mathematics, Institute of Biology, Ss. Cyril and Methodius University, 1000 Skopje, North Macedonia; nikola@pmf.ukim.mk (N.H.-P.);; 7 GloNeuro, Sector 107, Vishwakarma Road, Noida 201301, Uttar Pradesh, Indiajksinha@gloneuro.org (J.K.S.);; 8 Symbiosis Centre for Information Technology (SCIT), Rajiv Gandhi InfoTech Park, Hinjawadi, Pune 411057, Maharashtra, India; krishnakumar@scit.edu; 9 School of Management, BML Munjal University, NH8, Sidhrawali, Gurugram 122413, Haryana, India; 10 Department of Gastroenterology, All India Institute of Medical Sciences (AIIMS), New Delhi 110029, India; 11 School of Chemical Engineering, Yeungnam University, Gyeongsan 38541, Republic of Korea; 12 Research Institute of Cell Culture, Yeungnam University, Gyeongsan 38541, Republic of Korea

**Keywords:** oncogenes, tumor-suppressor genes, molecular pathways, tumor heterogeneity, tumor microenvironment, targeted cancer therapy, emerging technology, cancer research

## Abstract

Cancer, characterized by the uncontrolled proliferation of cells, is one of the leading causes of death globally, with approximately one in five people developing the disease in their lifetime. While many driver genes were identified decades ago, and most cancers can be classified based on morphology and progression, there is still a significant gap in knowledge about genetic aberrations and nuclear DNA damage. The study of two critical groups of genes—tumor suppressors, which inhibit proliferation and promote apoptosis, and oncogenes, which regulate proliferation and survival—can help to understand the genomic causes behind tumorigenesis, leading to more personalized approaches to diagnosis and treatment. Aberration of tumor suppressors, which undergo two-hit and loss-of-function mutations, and oncogenes, activated forms of proto-oncogenes that experience one-hit and gain-of-function mutations, are responsible for the dysregulation of key signaling pathways that regulate cell division, such as p53, Rb, Ras/Raf/ERK/MAPK, PI3K/AKT, and Wnt/β-catenin. Modern breakthroughs in genomics research, like next-generation sequencing, have provided efficient strategies for mapping unique genomic changes that contribute to tumor heterogeneity. Novel therapeutic approaches have enabled personalized medicine, helping address genetic variability in tumor suppressors and oncogenes. This comprehensive review examines the molecular mechanisms behind tumor-suppressor genes and oncogenes, the key signaling pathways they regulate, epigenetic modifications, tumor heterogeneity, and the drug resistance mechanisms that drive carcinogenesis. Moreover, the review explores the clinical application of sequencing techniques, multiomics, diagnostic procedures, pharmacogenomics, and personalized treatment and prevention options, discussing future directions for emerging technologies.

## Simple Summary

Proto-oncogenes and tumor-suppressor genes play important roles in preventing the development of tumors. The former supports healthy cell growth and division, while the latter regulates cell division. Mutations in proto-oncogenes, converting them into oncogenes, and mutations in tumor-suppressor genes can lead to uncontrolled cell proliferation and cancer development. This review explores key molecular pathways associated with these genes, highlighting their influence on cancer progression and treatment resistance, which are crucial for creating effective, personalized, and targeted treatments for patients. This comprehensive review also discusses the recent advancements and emerging technologies that enhance the analysis, diagnosis, prevention, and treatment of cancer at the genomic level.

## 1. Introduction

Cancer is defined as an abnormal growth of cells that can originate from any organ or body structure where immune cells are unable to destroy overly dividing cells [1]. Typically, cancer must reach a size of 1 cm or be comprised of 1 million cells before it can be detected and is then often labeled as a “mass”, “growth”, “tumor”, “nodule”, or “lesion” [1]. Most recent estimates provided by the World Health Organization (WHO)’s cancer agency, the International Agency for Research on Cancer (IARC), indicate that, in 2022, there were around 20 million new cases and 9.7 million deaths attributable to cancer [2]. Moreover, it was found that about one in five people will develop cancer in their lifetime, with approximately one in nine men and one in twelve women dying from the disease [2].

Since the inception of revolutionary screening tests and therapeutic interventions, clinicians and researchers have become quite proficient at classifying cancers and subsequently diagnosing them based on their distinct stages and morphology [3]. However, despite this, there have been challenges in identifying specific biological drivers that account for cancer heterogeneity. The genomic revolution offers a potential route to better understand the fundamental origins of various cancers by analyzing genomic profiles and identifying patterns of gene expression, genetic alterations, and mutation. These genetic patterns represent the sub-cellular mechanisms that are responsible for the development of cancer, and understanding them may lead to more precise and personalized approaches to cancer diagnosis and treatment [3]. Somatic mitochondrial DNA (mtDNA) mutations are another major contributor to tumorigenesis and have also been observed to influence apoptosis or controlled cell death, which further impacts the progression of cancer [4]. These mtDNA mutations tend to build up over time, eventually causing an inability for the DNA to repair itself and subsequently dysregulating cell proliferation [4]. While there is a functional understanding of mtDNA mutations, they often serve as secondary or synergistic contributors to cancer biology, along with driver genes, including both oncogenes and tumor-suppressor genes [5].

The main cancer-driver genes were sequenced several decades ago. However, there is little known about the significance of many variants and the interplay between multiple genes and other factors surrounding nuclear DNA damage [6]. Driver genes are genes in which mutations cause cancer to develop or progress, and their affiliated cancer-causing mutations are known as driver mutations [6]. One prevalent driver gene is the *TP53* gene, which encodes the tumor protein p53, a tumor suppressor that is essential for regulating cell division. Alterations in the p53 pathway are found in more than 50% of all human tumors [6]. Cancer-related genes can be classified into two categories: tumor suppressors, which normally inhibit cellular proliferation and promote apoptosis, and oncogenes, those that regulate cell proliferation and survival [7]. DNA repair genes, responsible for maintaining DNA repair and chromosome integrity, work together with these other driver genes to maintain cellular homeostasis, and their disruption can lead to tumorigenesis.

## 2. Molecular Mechanisms

### 2.1. Tumor-Suppressor Gene Inactivation and Oncogene Activation

Tumor-suppressor genes work to regulate cell division and promote controlled apoptosis under normal physiological conditions [8]. Dysfunction of these genes results in a loss-of-function (LOF) effect, subsequently causing uncontrolled cell growth—a hallmark of cancer [8]. The silencing of autosomal tumor-suppressor genes is explained through Alfred G. Knudson’s two-hit theory [8]. Knudson studied the molecular mechanisms of the *RB1* gene, which is responsible for retinoblastoma, and investigated its role in both hereditary and nonhereditary forms of the disease. In his research, Knudson proposed that cancer initiation requires two mutational events, where in the dominantly inherited form, one mutation is inherited via the germinal cells, and the second occurs in the somatic cells [9]. Meanwhile, in the nonhereditary form, both mutations occur in the somatic cells. Tumors are then initiated when these cells contain two damaged or “hit” alleles [9]. This mechanism involves LOF mutations followed by a loss of heterozygosity (LOH) at specific loci of the affected tumor-suppressor genes [8,10]. Heterozygosity refers to the presence of one normal and one abnormal allele, where the loss of the normal allele produces a locus with abnormal function, as observed in certain inherited autosomal dominant cancer susceptibility disorders [10,11]. If the altered gene is a tumor-suppressor gene, the LOH and its polymorphic markers leave only the abnormal allele, thus disabling the normal function of the gene and making the cell more susceptible to malignant transformation.

While tumor-suppressor genes are responsible for inhibiting cell division, proto-oncogenes are involved in four basic mechanisms that regulate normal cell growth: growth factors, growth factor receptors, signal transduction molecules, and nuclear transcription factors [12]. These regulators are essential for normal proliferation and differentiation, and when mutated, they can cause uncontrolled cell growth and neoplastic transformation [7,12]. The conversion of proto-oncogenes to oncogenes, also known as activation, involves gain-of-function (GOF) mutations and can be caused by a variety of mechanisms such as transduction, insertional mutagenesis, amplification, point mutations, and chromosomal translocations [8,12,13]. Unlike tumor-suppressor genes that require “two-hit” mutations for inactivation, only “one-hit” mutation can be sufficient to activate a proto-oncogene and cause carcinogenesis [14,15].

Transduction is the process by which retroviruses capture and insert a full or partial proto-oncogene from the host into their viral genome, which leads to deregulation [12]. The c-*SRC* proto-oncogene can be seen to be transduced into v-*Src* oncogene in the Rous sarcoma virus (RSV), causing it to lose its regulatory phosphorylation site. This loss results in unregulated tyrosine kinase activity and enhances the oncogenic potential, transforming the infected cells and forming tumors [12,16]. Insertional mutagenesis is a similar oncogene activator mechanism, as it involves the integration of a retrovirus’ promoter gene into its host’s genome near a proto-oncogene, resulting in its overexpression. An example of this process is the avian leukosis virus (ALV), which integrates upstream of the cellular myelocytomatosis oncogene (c-*MYC*), leading to an overexpression of the c-MYC transcriptional factor and the development of lymphomas [17,18]. In gene amplification, the creation of multiple copies of proto-oncogene leads to excessive protein production [17]. For example, excess c-*MYC* is observed in neuroblastoma, while human epidermal growth factor receptor 2 (*HER2*) amplification is linked to aggressive breast cancers [19,20]. Meanwhile, in point mutations, a single nucleotide change in a proto-oncogene can alter its amino acid sequence, creating overly activated proteins [17,21]. The most common example is the Ras gene family, where mutations at the 12, 13, or 61 codons impair GTPase activity, keeping Ras constantly active and promoting uncontrolled proliferation [17,22,23]. Chromosomal translocation occurs when a proto-oncogene is moved from its original location to a new chromosomal context, where it can become aberrantly regulated. A classic example of this is the formation of the Philadelphia chromosome in chronic myelogenous leukemia (CML) arising from a translocation between chromosomes 9 and 22, creating a fusion gene known as *BCR-ABL1* that results in upregulated tyrosine kinase activity [12,17,24].

### 2.2. Key Signaling Pathways Involved

Cell division is driven and regulated by complex molecular pathways designated to prevent uncontrolled cell proliferation. In the scope of cancer, the main mode of post-translational modifications comes in the form of protein kinases such as cyclin-dependent kinases (CDKs) and Polo-like kinases [25,26]. CDKs are a group of serine/threonine kinases that regulate cell division. They act as catalytic subunits and require association with proteins called cyclins to become fully active. This association forms a heterodimer complex, with the cyclins serving as regulatory subunits [27,28]. In human cells, there are 20 CDKs and 29 cyclins, where CDK1, CDK2, CDK3, CDK4, CDK6, and CDK7 directly regulate cell-cycle transitions and cell division, while CDK7-11 oversees gene transcription [29,30]. Moreover, cyclin-dependent kinase inhibitors (CKIs) are responsible for the negative regulation of the cell cycle in tandem with CDKs, working to prevent premature entry into the next phase of cell division [7,27,31].

**Figure 1:**
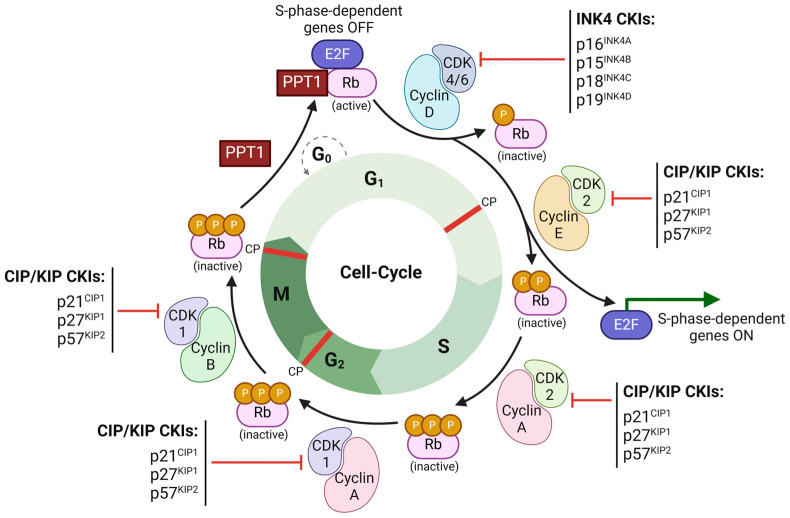
Schematic representation of cell-cycle regulation by cyclin-dependent kinases (CDKs), retinoblastoma protein (Rb), and cyclin-dependent kinase inhibitors (CKIs). Progression through the G_1_, S, and M phases is driven by the sequential activation of CDKs (CDK4/6, CDK2, and CDK1) in complex with their respective cyclins (cyclin D, E, A, and B), with G_0_ as a resting phase outside active cycling. The Rb protein, in its active, hypophosphorylated form binds E2F to block S-phase genes, aided by PPT1, until phosphorylation by CDK4/6-cyclin D and hyperphosphorylation by CDK2-cyclin E deactivates Rb, freeing E2F to turn S-phase-dependent genes on and thus trigger S-phase entry. There are three checkpoints (CP) that regulate the cell cycle: in G_1_, Rb halts progression if DNA damage is detected; in G_2_, CDK1-cyclin B blocks progression under stress or damage, ensuring proper timing for DNA repair; and in M, CDK1-cyclin B sustains mitosis until chromosome alignment is confirmed. INK4 inhibitors (p16^INK4A^, p15^INK4B^, p18^INK4C^, and p19^INK4D^) block CDK4/6 to prevent premature Rb phosphorylation and G_1_/S transition, while CIP/KIP inhibitors (p21^CIP1^, p27^KIP1^ and p57^KIP2^) block CDK2 and CDK1 to pause cycle progression under stress or damage, ensuring proper timing for DNA repair and checkpoint function. Created in BioRender. Stojchevski, R. (2025) https://BioRender.com/x80d496.

The resting phase, G_0_, is the state in which most cells are arrested prior to replication. Here, growth and arrest signals determine whether cells remain quiescent or enter the cell cycle (Figure 1) [7,30].

The transition from the G_0_ to G_1_ and G_1_ to S phase, where DNA replication occurs, is facilitated by the activation of CDK4 and CDK6 in complex with D-type cyclins (cyclin D1, cyclin D2, and cyclin D3) [32,33]. CDK4/CDK6-cyclin D complexes help push the cell past the restriction point in G_1_, committing to a new division cycle (Figure 1) [34]. These CDKs–cyclins complexes are regulated by the INK4 family of inhibitors, which include p16^INK4A^, p15^INK4B^, p18^INK4C^, and p19^INK4D^, which bind CDK4 and CDK6, preventing their association with D-type cyclins, thus suppressing their kinase activity [35]. As the cell progresses through G_1_, the CDK2–cyclin–E complex becomes active, further driving the cell’s progression into the S phase (Figure 1) [36]. During the S phase, CDK2 pairs with cyclin A to support replication, and later, CDK1 binds cyclin A in G_2_ and then cyclin B to initiate mitosis in the G_2_/M phase [33,37]. These CDK2 and CDK1 activities are modulated by the CIP/KIP family of inhibitors (p21^CIP1^, p27^KIP1^, and p57^KIP2^) to ensure proper progression or arrest (Figure 1) [35]. This complex regulation ensures that the process of cell division is well-controlled, preventing uncontrolled cell proliferation that may lead to cancer.

The CDK-cyclin complexes, particularly CDK4 and CDK6 with D-type cyclins, regulate the cell cycle by phosphorylating the retinoblastoma family of proteins Rb, p107, and p130, which act like tumor suppressors [32,33]. In its active, hypophosphorylated state, Rb binds E2F transcription factors, which inhibit the expression of genes required for the G_1_/S transition, such as cyclin E (*CCNE*), cyclin A (*CCNA*), thereby preventing premature cell-cycle progression in G_1_ [32,37,38]. On the other hand, when phosphorylated by CDK4/6 complexes, Rb loses its affinity for E2F, which allows E2F to activate these genes and drive the cell cycle into the S phase (Figure 1) [39]. Following mitosis, the Rb’s growth-suppressing function is restored through dephosphorylation by protein phosphatase 1 (PP1), returning it to its active, hypophosphorylated state [40].

Dysregulation of the Rb pathway, often through mutations in the *Rb1* gene, leads to uncontrolled E2F activity, resulting in aberrant cell proliferation and tumorigenesis. In human cancers, the key regulators of G_1_ phase, such as CDK4/6, their positive regulator cyclin D1, negative regulators p16^INK4A^ and p15^INK4B^, and their substrate Rb, are frequently altered [33]. Amplification of cyclin D1 or CDK4, translocation of CDK6, and deletions of INK4 proteins are key events disrupting cell-cycle control and contributing to carcinogenesis [33]. The CDK4/6-mediated phosphorylation is essential for the normal cell division process, and its hyperactivation results in excessive Rb protein phosphorylation, impairing its tumor-suppressor abilities and contributing to uncontrolled proliferation [33,41]. Recent findings by Zhang et al. [42] suggest that Rb can also be deactivated independently of CDK phosphorylation, such as through altered upstream signaling pathways or viral oncoprotein interactions, further inducing LOF mutations and nullifying its tumor-suppressor capabilities.

Regarded as the “guardian of the genome”, understanding the inactivation of the *TP53* tumor-suppressor gene is crucial, as it is mutated in over 50% of human cancers and in the other 50% is compromised through biological inactivation by its negative regulators like murine double minute 2 (MDM2) and X (MDMX) or through mutations in upstream kinases [43]. Under normal conditions, p53 acts as a transcription factor that induces the expression of *CDKN1A* (p21), a cyclin-dependent kinase inhibitor that blocks CDK4/6 and CDK2 to maintain the active hypophosphorylated state of Rb [44,45]. This allows Rb to form a complex with E2F, thus blocking the G_1_ phase and enforcing cell-cycle arrest by suppressing the genes required for S-phase entry [45,46]. MDM2, an E3 ubiquitin ligase, and MDMX, its cofactor, negatively regulate p53, targeting it for ubiquitination and degradation by proteasomes in the cytoplasm [44,47,48]. During cellular stress like DNA damage, the ubiquitination of p53 is inhibited, triggering a rapid increase in intracellular p53 levels. This enables p53 to promote the expression of genes for apoptosis, cell-cycle arrest, or DNA repair, contributing significantly to its tumor-suppressor role [49,50,51,52].

The genomic amplification of the *MDM2* locus, located on chromosome 12q15 in humans, is a common alteration in most cancers [43,53]. This overexpression of the MDM2 protein is often caused by chromosomal aberrations such as gene duplication, aneuploidy, or focal amplification [54,55]. Upregulation of *MDM2* transcription may also result from oncogenic signals, such as epidermal growth factor (EGF) and insulin-like growth factor-1 (IGF-1), which activate the PI3K/AKT and MAPK pathways and phosphorylate MDM2 at residues Ser166 and Ser186 [56,57,58,59]. MDM2 amplification has broad consequences, primarily in the rapid ubiquitination and proteasomal degradation of p53, thereby effectively silencing p53-mediated transcriptional programs and neutralizing its tumor-suppressor properties [44,60]. Furthermore, MDM2 amplification downregulates p21, which allows cyclin–CDK complexes to hyperphosphorylate Rb at serine and threonine residues [45,61,62]. This hyperphosphorylation causes conformational changes in Rb, disrupting its ability to inhibit E2F, which ultimately accelerates cell-cycle progression [33,41]. Additionally, MDM2 amplification impairs p53-regulated processes, such as apoptosis and genomic stability. p53 is known to activate pro-apoptotic genes such as *BAX*, *NOXA*, and *PUMA*, in which NOXA and PUMA act as upstream regulators that inhibit anti-apoptotic proteins, enabling BAX to permeabilize the mitochondrial membrane and trigger apoptosis [44,63,64,65,66]. Moreover, p53 maintains genomic stability by inducing growth arrest and DNA damage-inducible 45 (*GADD45*) and various DNA repair enzymes. Therefore, MDM2 amplification, by inhibiting p53, results in mutation accumulation and promotes tumorigenesis [67].

**Figure 2:**
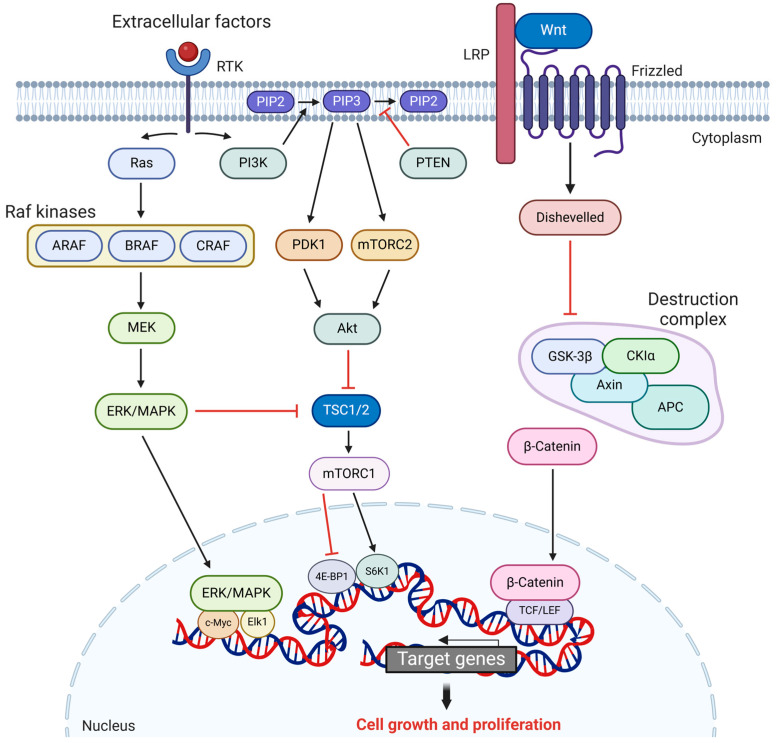
Key oncogenic signaling pathways. Left: The RAS/ERK/MAPK pathway is initiated when an RTK receptor is activated by extracellular signals, leading to the activation of Ras. Activated Ras recruits Raf kinases (ARAF, BRAF, CRAF/RAF1) to the cell membrane, where they phosphorylate MEK at Ser166 and Ser186. MEK then phosphorylates and activates ERK/MAPK. Activated ERK/MAPK translocates to the nucleus, where it activates transcription factors Elk1-1 and c-MYC, thus promoting cell proliferation. Middle: The PI3K/AKT pathway, like the RAS/ERK/MAPK pathway, is activated through RTK receptors. Activated RTK triggers PI3K, which converts PIP2 into PIP3. PIP3 provides a docking site for PDK1 and mTOR2, which phosphorylate and activate AKT, which then phosphorylates and inhibits TSC2, a negative regulator of mTORC1. This inhibition indirectly activates mTORC1, which promotes cell growth by modulating transcription factors 4E-BP1 and S6K1, key regulators of protein synthesis. Right: The Wnt/β-catenin begins when Wnt ligands bind to the frizzled receptor and LRP co-receptors, activating the Dishevelled protein. Dishevelled inhibits the destruction complex (composed of GSK-3β, Axin, CK1α, and APC), preventing the degradation of β-catenin. This allows β-catenin to accumulate and translocate to the nucleus, where it activates transcription factors TCF and LEF, driving cell proliferation. Created in BioRender. Stojchevski, R. (2025) https://BioRender.com/p57k526.

Oncogenes follow signaling pathways that operate both independently and in conjunction with those of tumor-suppressor genes. While tumor suppressors primarily follow the p53 and Rb pathways, one of the most common oncogenic events is the dysregulation of the Ras/Raf/MEK/ERK MAPK pathway (Figure 2) [68]. The genes in the Ras oncogene family are among the first oncogenes to be discovered forty years ago, and mutations in this pathway prevail in 25% of all tumors [69]. The pathway begins when receptor tyrosine kinases (RTKs) are activated by extracellular signals, causing them to activate guanine nucleotide exchange factors (GEFs) and, subsequently, Ras, a family of small GTP-binding proteins (HRAS, KRAS, and NRAS) [70,71]. Activated Ras then recruits Raf kinases to the cell membrane, specifically ARAF, BRAF, and CRAF (also known as RAF-1), which are serine/threonine kinase enzymes and isoforms of each other [72,73]. BRAF is the most active of these kinases and has a role in the phosphorylation of mitogen-activated protein kinase kinase (MEK) at Ser166 and Ser186, which then activates mitogen-activated protein kinase (MAPK), also known as extracellular signal-regulated kinase (ERK) [73,74,75].

The regulation of MAPK/ERK by MEK is a crucial intracellular process that allows ERK to translocate to the nucleus, where it regulates the activity of key transcription factors, including Elk-1 and c-MYC, promoting cell proliferation [68,73,76]. Dysregulation of this pathway is a major driver of oncogenic events, particularly through mutations in the *KRAS* and *BRAF* genes. *KRAS* mutations are a predominant factor in the activation of the MAPK pathway in tumors. These mutations are characterized by single-base missense changes, with approximately 98% occurring at codons 12 (G12), 13 (G13), or 61 (Q61) [77]. These alterations lock *KRAS* in an active GTP-bound state, continuously signaling via the MAPK and PI3K pathways to promote uncontrolled cell growth and survival. Recent developments have introduced *KRAS* G12C-specific inhibitors, such as sotorasib and adagrasib, that effectively target and bind the mutant protein, blocking its activity and offering promising therapeutic advances [78]. The *BRAF* V600E mutation, prevalent in melanomas, and also observed in thyroid, ovarian, and lung cancers, occurs when valine (V) is replaced by glutamic acid (E) at position 600 [72,79,80]. This mutation forces a constant Ras-independent activation of ERK/MAPK, thus promoting uncontrolled cell migration and proliferation [81]. Furthermore, hyperactivation of this pathway leads to the overexpression of anti-apoptotic genes like B-cell lymphoma-2 (*Bcl-2*), decreasing pro-apoptotic signals and allowing cell survival in tumor microenvironments despite various stresses [79,80]. Moreover, the *BRAF* V600E mutation increases vascular endothelial growth factor (VEGF) expression as a downstream effect, facilitating invasion by activating matrix metalloproteinases (MMPs) and, thus, inducing angiogenesis [82,83].

Much like the MAPK pathway, the phosphoinositide 3-kinase (PI3K)/AKT pathway is crucial for regulating cellular growth, survival, and proliferation (Figure 2) [84]. This pathway is activated when RTKs trigger PI3K, which then converts phosphatidylinositol-4,5-bisphosphate (PIP2) into phosphatidylinositol-3,4,5-trisphosphate (PIP3), providing a docking site for 3-phosphoinositide-dependent protein kinase 1 (PDK1) and mTOR complex 2 (mTORC2), which then phosphorylate and activate protein kinase B (AKT) [85]. AKT is a serine/threonine kinase similar to the Raf family, which phosphorylates several proteins with different outcomes. It activates the mechanistic target of rapamycin (mTOR), a kinase that regulates protein synthesis; inhibits Bcl-2-associated death promoter (BAD), a pro-apoptotic protein; and inhibits forkhead box O (FOXO), a family of transcription factors that promote apoptosis and cell-cycle arrest [86,87,88,89]. mTOR signaling is modulated by the PI3K/AKT pathway through the phosphorylation of its negative regulator, TSC2, which indirectly activates mTORC1 [90,91]. This activation of mTORC1 leads to a subsequent cascade in downstream pathways such as 4E-BP1 and S6K1, which are both involved in mRNA translation and protein synthesis [92]. Disruption of the PI3K/ATK pathway can occur from mutations in *PIK3CA*, the PI3K catalytic subunit, or by the loss of phosphate and tensin homolog (PTEN), a tumor suppressor that dephosphorylates PIP3 to inhibit AKT activation, resulting in oncogenesis through uncontrolled proliferation, metabolic reprogramming, resistance to apoptosis, and GOF missense mutations [89,93]. While disruption of the PI3K/ATK pathway significantly contributes to oncogenesis, its effects are further amplified by extensive crosstalk with the MAPK pathway [94]. Both signaling pathways share RTKs as activators, creating a feedback loop that interconnects them, where ERK can activate mTOR and mTORC1 modulates MAPK signaling [91,95]. As a direct result, failure in either or both mechanisms causes oncogenesis through rapid cell division.

The Wnt/β-catenin is another crucial signaling pathway that governs cell proliferation, differentiation, and stem-cell maintenance (Figure 2) [96]. In the absence of Wnt ligands, the destruction complex, consisting of adenomatous polyposis coli (APC), glycogen synthase kinase-3β (GSK-3β), and Axin, is active, which is responsible for the phosphorylation of β-catenin, targeting it for degradation [96,97,98,99]. When Wnt ligands bind to a frizzled receptor and LRP5/6 co-receptors on the cell surface, this activates the recruitment of the Dishevelled protein (DVL), which deactivates the destruction complex, thus inhibiting the degradation of β-catenin. Accumulated β-catenin then translocates in the nucleus, activating transcription factors like TCF and LEF and promoting cell proliferation [97,100]. Wnt/β-catenin is most often disrupted through mutations in catenin-Beta-1 (*CTNNB1*), which encodes β-catenin, or in *APC*, subsequently reducing β-catenin degradation and leading to persistent nuclear localization and activation of Wnt target genes [101,102]. Moreover, dysregulation of the Wnt pathway promotes cancer metastasis by inducing epithelial-to-mesenchymal transition (EMT), where β-catenin activates transcription factors like Snail and Slug, leading to downregulation of epithelial markers (e.g., E-cadherin) and upregulation of mesenchymal markers (e.g., vimentin and N-cadherin) [96,103,104]. The Wnt/β-Catenin pathway also exhibits crosstalk with MAPK and PI3K, causing a synergistic effect of oncogenesis and metastatic development [105]. Wnt signaling can upregulate PI3K/AKT activity by increasing PIP3 levels via downstream targets or modulating PTEN expression to enhance cell survival and metabolism [106,107]. Conversely, AKT can stabilize β-catenin by phosphorylating and inhibiting GSK-3β, thus amplifying Wnt signaling [108,109]. Similarly, the MAPK pathway interacts with Wnt signaling, where β-catenin can influence Ras activity and Raf/MEK/ERK signaling downstream of Ras to promote proliferation, making these three pathways the most significant and prominent players in the processes of oncogenesis [110,111].

### 2.3. Epigenetic Regulation

Epigenetics is modernly defined, according to Sharma et al. [112], as the study of heritable changes in gene expression that occur independently of changes in the primary DNA sequence. Disruption of these epigenetic processes can result in inappropriate activation or inhibition of various signaling pathways that alter gene functions and cause malignant cellular transformation [113,114]. DNA methylation, a critical epigenetic process, involves the addition of methyl groups to specific cytosine residues at CpG sites, regulating gene expression by silencing certain genes and suppressing repetitive genomic elements [115,116,117]. This process is regulated by DNA methyltransferases (DNMTs), which alter chromatin’s structure to modulate expression [118]. Aberrant DNA methylation is a hallmark of carcinogenesis, as irregular methylation leads to the silencing of tumor-suppressor genes and the induction of oncogenesis [116]. Hypermethylation and global hypomethylation are the two types of aberrant DNA methylation that affect tumor suppressors and proto-oncogenes, respectively [119]. DNA promoter hypermethylation is often an early event in tumorigenesis and is commonly observed in tumor-suppressor genes, such as cyclin-dependent kinase inhibitor 2A (*CDKN2A*), breast cancer gene 1 (*BRCA1*), and MutL protein homolog 1 (*MLH1*), among others, where excessive methylation represses transcription, ultimately disabling cell-cycle barriers that normally limit cell proliferation and compromising the cellular functions that are crucial for maintaining genomic stability and repair [120,121,122,123]. On the other hand, global hypomethylation refers to a decrease in methylation across the genome, which in repetitive sequences can lead to chromosomal rearrangements, translocations, and aneuploidy, facilitating proto-oncogene activation [124,125]. The most notable example of global hypomethylation in proto-oncogenes is the activation of the Ras family genes, particularly the H-Ras proto-oncogene, where hypomethylation leads to its upregulated expression through long-range interactions between distant chromatin regions, allowing enhancers to activate the H-Ras promoter more effectively [126]. Recent studies have also shown that hypermethylation and hypomethylation can coexist in cancers, resulting in a dual mechanism that amplifies the accumulation of aberrant DNA mutations in both tumor suppressors and oncogenes [127,128].

Histone modifications and chromatin remodeling are another group of essential epigenetic processes whose malfunction can contribute to carcinogenesis [129]. Histones are small, basic proteins rich in arginine and lysine residues, around which DNA wraps itself to form nucleosome cores, leading to the formation of chromatin and, ultimately, mitotic chromosomes [130,131]. Post-translational modifications of histones include acetylation, methylation, phosphorylation, and ubiquitination, which play a role in regulating chromatin dynamics and gene expression [132]. Aberrant histone modifications disrupt chromatin accessibility, causing dysregulation of both tumor-suppressor genes and oncogenes [129]. For instance, the *BRCA1* tumor-suppressor gene, which can undergo DNA hypermethylation, is also found to be silenced in breast and ovarian cancers due to histone deacetylation mediated by histone deacetylases (HDACs) [132,133]. Moreover, trimethylation of histone H3 at the lysine 27 position (H3K27me3), catalyzed by polycomb-repressive complex 2 (PRC2) subunit EZH2, has been found to silence tumor-suppressor genes such as *INK4a*/*ARF* [134,135]. These aberrant histone modifications have also been linked to oncogenesis, as exemplified by H3K4me3, which promotes the transcription of oncogenes, including *CCND1* and *MYC* [136].

MicroRNAs (miRNAs) are small noncoding RNAs that regulate gene expression at the posttranscriptional level, and, like DNA methylation and histone modifications, their abnormal expression can lead to cancer progression [133]. miRNAs exhibit pleiotropic functions, acting differently depending on their cellular and tissue-specific context [137]. The first miRNAs to be associated with cancer, miR-15 and miR-16, were identified in B-cell leukemia, and since then, strong links have been established between the biogenesis of miRNA and their target genes, including tumor suppressors and oncogenes [133,138]. These miRNAs have been found to play a critical role in the regulation of the key signaling pathways involved in cell proliferation. For instance, miR-99a has been found to inhibit the target of mTOR, whereas miR-19 and miR-501-5p activate the Wnt/β-catenin signaling pathway [139,140]. Additionally, miR-21 and miR-221/222 stimulate the development of cancer stem cells through the inhibition of PTEN [141]. Even more, the Let-7 family of miRNAs has major implications in other pathways, such as activating Ras oncoproteins and deregulating signal transduction pathways, including PI3K/AKT [142]. The interplay of DNA methylation, histone modifications, and miRNA expression creates a complex epigenetic network that controls the silencing of tumor suppressors and the activation of oncogenes. These epigenetic alterations do not simply drive the development and progression of cancers but also hold great promise for therapeutic intervention.

## 3. Challenges and Controversies

### 3.1. Tumor Heterogeneity

Due to rapid mutation rates during cell proliferation, cancer is characterized by its ability to evolve in composition over time. Throughout the progression of the disease, cancer becomes increasingly heterogeneous in nature, developing subsets of genetically distinct cell populations. These populations can exhibit variations in cell surface markers, levels of expression of tumor-driver genes, and copy number alterations depending on the tumor microenvironment [143]. Tumor heterogeneity can be observed both within a singular tumor, referred to as intratumoral heterogeneity, and between a primary tumor and its distant metastases, known as intertumoral heterogeneity [144]. As a heterogeneous tumor accumulates mutations and chromosomal aberrations, it gains the potential to resist pharmacological treatment, leading to poor clinical outcomes and tumor relapse [144,145]. A multiregional sampling study conducted by Jamal-Hanjini examined one hundred early-stage non-small cell lung cancer (NSCLC) tumors and found that a median of 30% of somatic mutations were subclonal in nature, indicating a high level of tumor heterogeneity [146]. The study determined that more extensive copy number heterogeneity is associated with worse clinical outcomes in patients, as it is representative of underlying genomic instability [144].

Proposed models for the evolution of intratumoral heterogeneity offer insight into how cancers continue to persist despite treatment. Evidence shows that many solid tumors undergo a branched pattern of evolution, wherein multiple different subclonal populations arise from a common ancestor [147]. These subclonal populations emerge simultaneously and do not completely outcompete and replace the preceding clones. Instead, the clones coexist in a mosaic-like pattern. This dynamic process allows the tumor to develop a higher degree of heterogeneity, increasing its chance of survival against the selective pressures imposed by pharmacologic treatment [144]. High rates of intratumoral heterogeneity are associated with many malignant and aggressive cancers. For example, a single-cell sequencing study conducted by Shi et al. found that recurrent bladder cancer tumors displayed greater local cell-type heterogeneity than primary bladder cancer tumors [148]. The recurrent tumors exhibited more complex immune and stromal cell populations, differing in composition from the primary tumor, thus contributing to their ability to interact with the immune response [148]. A similar pattern was observed in multi-region biopsies of medulloblastoma, malignant glioma, and renal cell carcinoma, where genes targeted for therapy were highly heterogeneous within tumor samples [149]. Morrisy et al. [149] argue that monotherapies aimed at a single target are unlikely to be effective, as the target is not ubiquitously present, allowing the untargeted clones to remain and repopulate the tumor. The presence of multiple distinct cell lineages within a single tumor has been linked to poor prognosis in patient outcomes, highlighting that intratumoral heterogeneity poses great challenges for targeted therapies [150].

Intertumoral heterogeneity between a primary tumor and its metastases develops in a similar manner to intratumoral heterogeneity, but the evolution is markedly shaped by the tumor microenvironment of the metastases. The tumor microenvironment includes all of the unique conditions under which a primary tumor or its metastasis develops. Tumor micro-environments may differ in their vasculature, susceptibility to immune attack, and other growth conditions, each creating specific challenges and opportunities for cell populations to thrive and evolve [151]. For instance, it has been shown that a tumor microenvironment with disordered blood vessel formation leads to an uneven distribution of cancer therapy drugs, thus leading to the proliferation of drug-resistant clonal populations [152]. The tumor microenvironment can differ vastly from site to site, explaining why intertumoral heterogeneity can be observed in both local and distant metastasis sites. In the case of clear-cell renal cell carcinoma, local metastasis is initiated with the formation of a tumor thrombus in the proximal vasculature [153]. Evidence shows that this thrombus originates from advanced subclonal cells of the primary tumor, which exhibits a loss of the 9p chromosome, a significant marker for tumor progression and malignancy that leads to poor prognosis in this type of cancer [154,155]. Intertumoral heterogeneity between a primary tumor and distant metastases is also common and can be explained by polyclonal seeding, in which different metastatic sites are established by genetically distinct subclones that originated from the same primary tumor [144]. The metastases are then subjected to the selective pressures presented by the respective tumor microenvironments in which they develop [144].

### 3.2. Drug-Resistance Mechanisms

Clonal evolution poses significant challenges in the pharmacological treatment of cancer due to the acquisition of mutations that confer drug resistance. Chemotherapy presents yet another selective pressure that drives clonal evolution, ultimately resulting in a population of multidrug-resistant cancer cells. A key mechanism of resistance relies on the multidrug resistance-associated proteins (MRPs), a family of transmembrane efflux pumps within the ATP-binding cassette (ABC) transporter group [156]. MRPs utilize the energy from ATP hydrolysis to expel cytotoxic drugs from cancer cells, decreasing the intracellular drug concentration and diminishing the general chemotherapeutic effect [157]. MRP1, MRP3, MRP4, and MRP5, for instance, have often been found to be overexpressed in gastric adenocarcinoma (GAC), a highly chemoresistant cancer driven by oncogene activation, particularly through the upregulation of efflux pumps via the EGFR, Ras, or MYC pathways [158,159,160]. In a study by Al-Abdulla et al., inhibition of MRP1 and MRP4 using drugs like diclofenac and probenecid increased the responsiveness of GAC to chemotherapeutic agents [160]. These findings indicate that targeting MRP efflux pumps with specific inhibitor drugs can improve the efficacy of chemotherapy. Breast cancer resistance protein (BCRP) is another MRP within the ABC transporter superfamily, which transports substrates, including chemotherapeutic agents such as mitoxantrone and methotrexate [161]. Although the chemotherapeutic treatment is initially successful, its efficacy diminishes as drug resistance develops through clonal evolution. However, a study has found BCRP to be overexpressed in chemotherapy-naive breast cancer brain metastases, indicating intrinsic drug resistance properties [162].

Cancers have also been shown to upregulate key DNA repair mechanism enzymes, therefore decreasing the efficacy of chemotherapeutic drugs that aim to disrupt the S phase of the cell cycle. One such enzyme is DNA polymerase theta (POLQ), which repairs double-stranded DNA breaks through microhomology-mediated end-joining [163]. *POLQ* is overexpressed in cancer cells with deficient repair mechanisms to fill single-stranded gaps, allowing continued replication and proliferation of cancer cells [163]. Moreover, POLQ is found exclusively in cancer cells, suggesting that it acts as a compensatory mechanism that ensures cancer cell survival [163]. Multiple studies suggest that upregulation in *POLQ* is associated with poor prognosis in patients with breast cancer, with around a 4-fold increased risk of death when the enzyme is overexpressed [164,165]. Similarly, another key regulatory enzyme associated with the repair of single-stranded breaks (SSBs), poly (ADP-ribose) polymerase 1 (PARP1), has been found to be overexpressed in multiple cancers. It rapidly detects and binds to SSBs, playing a vital role in the activation of DNA polymerases and ligases to fix these lesions [166]. Furthermore, nuclear PARP1 overexpression has been observed in various breast cancers, including ductal and highly malignant triple-negative breast carcinomas, and even in breast cancer stem cells [167,168,169]. PARP1 is key for maintaining genomic stability and facilitating DNA repair. Thus, PARP1 overexpression promotes the upholding of the cancer integrity and its continued proliferation. Consequently, high levels of nuclear PARP1 correlate with poor prognosis, contributing to lower disease-free and overall survival rates in breast cancer patients [170]. Additionally, *PARP1* overexpression has also been observed in recurrent oral cancers that have developed resistance to chemotherapy and radiation, linking PARP1 function to progressed malignancies [171]. In *BRCA1*-deficient cells, POLQ and PARP play important roles in DNA repair, with POLQ mediating error-prone repair via microhomology-mediated end-joining (MMEJ), while PARP1 addresses single-strand break repair. Simultaneously targeting both *POLQ* and *PARP1* may increase synthetic lethality, leading to improved therapeutic effects by impairing compensatory repair mechanisms [172,173].

Cancers have also been found to have an increased expression of glutathione, a powerful antioxidant that aids in cancer’s resistance to chemotherapeutic drugs designed to induce cytotoxicity via reactive oxygen species (ROS) [174]. Glutathione, a tripeptide containing cysteine and sulfur, exists predominantly in its reduced form, enabling it to bind and neutralize ROS. This antioxidant role is particularly important in cancer cells, where ROS levels are generally elevated due to the extensive metabolism needed for cell proliferation [175]. Upregulated glutathione function has been observed in lung, breast, ovarian, and head and neck cancers, with cancer cells often expressing nearly ten times the glutathione levels of healthy cells [176,177]. Although prooxidant chemotherapies have been developed to increase ROS production and induce tumor cell death, their effectiveness is limited due to the tumor cells’ ability to mitigate excessive oxidative stress [178]. The overexpression of glutathione may be linked to the deregulation of the RAS/MAP signaling pathway due to mutations in the genes activating KRAS [179]. For example, a study on lung cancer tumors found that cells with mutant KRAS exhibited increased channeling of metabolites into glutathione biosynthesis, enhancing their antioxidant capacity [179]. Similarly, colorectal cancer patients with KRAS mutations exhibited increased glutathione levels, which suppressed ferroptosis, an iron-dependent form of cell death triggered by ROS and lipid accumulation [180,181]. Moreover, KRAS mutations have been shown to alter amino acids, fatty acids, and nucleotide biosynthesis in cancer cells, further highlighting the role of oncogenic mutations in promoting drug resistance across various tumor types [182].

### 3.3. The “Gray” Area Between Oncogenes and Tumor-Suppressor Genes

The traditional classification of oncogenes and tumor-suppressor genes has provided a basis for targeted cancer therapies. However, current studies reveal that certain genes can exhibit both oncogenic and tumor-suppressive roles depending on specific conditions, thus complicating the development of effective therapeutic interventions [183]. Oncogenic transformations that drive uncontrolled cell proliferation can also affect tumor suppressors. For example, while the *TP53* gene is inherently a tumor suppressor, it can also undergo GOF mutations (e.g., R175, R248), acquiring oncogenic properties independent of its LOF effects [184]. Mutations in six “hot spot” amino acids of its coding sequence have been linked to increased proliferation, inhibition of apoptosis, enhanced inflammation, and the induction of angiogenesis [184,185]. Conversely, uncontrolled *MYC* expression can trigger an oncogenic-stress response via MDM2 to stimulate p53-mediated apoptosis, mimicking tumor-suppressor behavior [186]. Similarly, transforming growth factor beta 1 (*TGFβ1*) normally acts as a tumor suppressor by inhibiting epithelial growth and promoting apoptosis [187], but pathway defects (e.g., SMAD loss) can lead to a cascade of downstream events that drive multiple oncogenic processes like sustained angiogenesis, loss of apoptosis, and immune evasion [187]. These examples, summarized in Table 1, illustrate how context-dependent conditions and mechanisms, such as mutation-induced protein changes, stress responses, and disrupted signaling, drive these shifts and reshape gene function. Recognizing the complexities involved in this area deepens our understanding of cancer pathogenesis and emphasizes the need for personalized therapeutic approaches that are capable of addressing mutations at the genomic level within tumor heterogeneity

## 4. Clinical Implications

### 4.1. Diagnostic Approaches

Next-generation sequencing (NGS) is a modern tool in genomics research that can rapidly sequence millions of DNA fragments simultaneously [188]. This transformative technology is highly regarded for its high throughput and cost-effectiveness in analyzing DNA and RNA, producing unparalleled comprehensive insights into genome structure, genetic variations, gene expression profiles, and epigenetic modifications [188,189]. NGS techniques significantly differ from the widely used traditional Sanger sequencing approach [188]. The Sanger method, named after its creator Fred Sanger, was a revolutionary DNA sequencing technique that involved the separation and analysis of DNA strands using gel and later capillary electrophoresis [190]. While it was largely considered the gold standard in genomics research and was used in the Human Genome Project, the technique eventually reached its ceiling due to its ability to analyze only one sequence at a time, thus limiting its scalability and efficiency [191]. These limitations of the Sanger method paved the way for the development of NGS, where techniques such as whole-genome sequencing (WGS), whole-exome sequencing (WES), and epigenome or targeted sequencing (TS) can achieve more comprehensive results using less time and fewer resources [191,192,193,194].

WGS is an application of NGS used to determine the complete DNA sequence of an organism’s genome at once. WGS covers coding (exons) and noncoding (introns) sequences, circulating DNA, copy number variations, and genomic structural rearrangements [188,195]. By sequencing the entire genome, WGS enables the identification of genetic variations, such as single-nucleotide polymorphisms (SNPs), and structural changes, like insertions and deletions [188]. WGS utilizes two sequencing approaches: short-read and long-read protocols [196,197]. Short-read protocols produce reads of less than 300 base pairs (bp), whereas long-read protocols can range from 1000 bp or kilobase pair (kbp) to several megabase pairs (mbp) [196]. Both protocols hold their merits as short-reads are especially useful for providing high accuracy and depth for detecting smaller variants at a low cost per base, while long-reads improve sequence phasing and are better suited for identifying more significant haplotypes and complex structural variants [198,199]. This technology is especially promising when applied to newborn screening, cancer detection, genetic diseases, and personalized medicine [200]. WGS finds its primary clinical applications in diagnosing rare diseases and identifying actionable somatic variants in tumors, making it especially useful for studying the activation of proto-oncogenes and the silencing of tumor-suppressor genes at the molecular level [199,201]. A recent study by Kinnersley et al. [202] showed the extensive potential of WGS for tumor genomic profiling, where they sequenced data from 10,478 patients spanning 35 different cancer types, identifying 330 candidate-driver genes, of which 74 were newly associated with cancer.

Similar to WGS, WES is an efficient strategy for selectively sequencing the coding regions of a genome to discover variants [188]. The method captures the protein-coding regions of the genome, known as the exome, and identifies single-nucleotide variants (SNVs), insertions, deletions, and copy number variations (CNVs) within these genes [203,204]. Regions outside this focus, such as introns, promoters, and intergenic regions, are not sequenced [205]. Like WGS, WES has extensive clinical applications, particularly in cancer genomics [206]. WES is crucial for detecting highly susceptible genes, offering opportunities for preventive measures [206]. A study by Hamdi et al. [207] performed WES on seven Tunisian breast cancer families to detect shared genetic variations that may enable early intervention strategies, identifying four novel candidate genes associated with breast cancer risk: *MMS19*, *DNAH3*, *POLK,* and *KAT6B*. In reference to the “two-hit theory”, this early screening could enable the detection of an inherited mutated allele, providing a window for therapeutic strategies. WES has also been valuable in post-cancer diagnosis and during treatment phases, where it can provide comprehensive genetic profiling of tumors and help identify targetable mutations in oncogenes and tumor suppressors [206].

TS is the third technique stemming from NGS, and while WGS and WES focus on comprehensive and broad coverage, TS panels focus on very specific genomic regions, requiring less computational demand [208,209]. Like other NGS methods, TS can detect genetic variations, including SNVs, small gene deletions, duplications, insertions, and rearrangements, but on a smaller scale, making it more cost-effective and easier for clinicians to manage [188,209]. TS offers greater sequencing depth for various tumor contents, such as circulating tumor DNA (ctDNA) and formalin-fixed paraffin-embedded (FFPE) samples, compared to non-NGS-based techniques like Sanger sequencing and allele-specific PCR (AS-PCR) [208]. Like WES, TS is particularly useful in the post-cancer phase, as it can isolate the mutations present in a small portion of malignant cells and detect variant allele frequency (VAF) as low as 0.1–0.2% [210,211,212]. Moreover, TS can identify any minor aberration of tumor-suppressor genes that may be clinically significant, highlighting its potential for guiding treatments targeting damaged tumor-related genes.

### 4.2. Therapeutic Strategies

**Figure 3:**
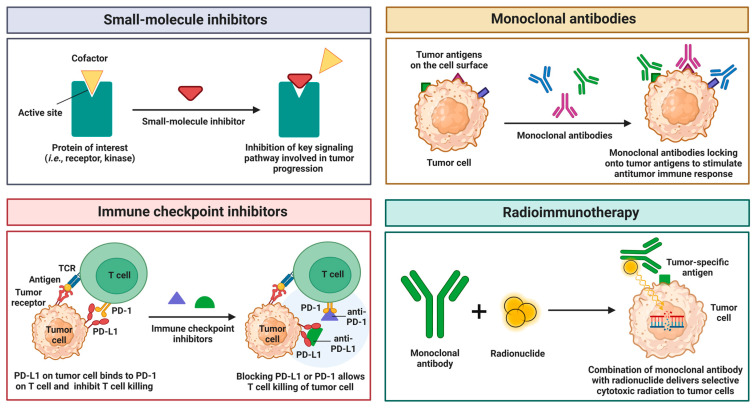
Mechanisms of action of small-molecule inhibitors, monoclonal antibodies, immune checkpoint inhibitors, and radionuclides in cancer therapy. Small-molecule inhibitors target key receptors and kinases to disrupt signaling pathways and block tumor progression. Monoclonal antibodies recognize and bind specific antigens on tumor cell surface, leading to immune-mediated destruction. Immune checkpoint inhibitors enhance T-cell activity by blocking inhibitory immune signals, restoring the immune system’s ability to recognize and eliminate tumor cells. Radioimmunotherapy combines monoclonal antibodies with radionuclides to selectively deliver cytotoxic radiation to tumor cells, increasing treatment precision while minimizing damage to normal tissues. Created in BioRender. Stojchevski, R. (2025) https://BioRender.com/k49h851.

As diagnostic approaches advance, so do the therapeutic strategies designed to combat the genomic changes that drive tumorigenesis. Among the most promising novel techniques is targeted therapy, which is a form of precision medicine that targets the specific proteins and genetic changes responsible for tumor heterogeneity [213]. The key advantage of targeted therapy is its ability to affect only abnormal proteins, unlike traditional chemotherapy, which is nonselective and can be indiscriminately toxic to all cells [213,214]. Targeted therapy includes various approaches, including immunotherapy, small-molecule inhibitors, and monoclonal antibodies (Figure 3) [215,216,217].

Small-molecule inhibitors are broadly used in targeted therapy designed to slow or kill tumor cells by primarily targeting protein kinases, which are highly active pro-growth signaling initiators [218]. Their low molecular weight allows them to diffuse through cells and target intracellular drivers that regulate proliferation and apoptosis [214]. The list of various U.S. Food and Drug Administration (FDA)-approved small-molecule inhibitors for cancer treatment is shown in Table 2.

The predominant and widely accepted class of small-molecule inhibitors includes those targeting RTKs and VEGF receptors, such as Erlotinib, Sunitinib, and others, which exert antiangiogenic and antiproliferative effects [219]. Recent breakthroughs have shown promising applications of small-molecule inhibitors in treating oncogene-driven mutations [214]. Serval inhibitors for BRAF of the MAPK pathway, such as Vemurafenib and Dabrafenib, have shown effective results against melanomas. Moreover, these inhibitors are particularly potent in treating patients with Ras and BRAF V600E mutations when used in combination with general MAPK inhibitors like Trametinib [214,220]. Despite their promise, small-molecule inhibitors have limitations, as they lead to the development of drug resistance through mechanisms that may include their influence on tumor microenvironments and the potential reactivation of both MAPK and PI3K/AKT signaling pathways [220,221]. Additionally, resistance may arise from the changes occurring in the genes coding for target proteins, deviation in signaling pathways that activate different proteins with similar functions, or mutations in the genes coding for the proteins associated with the target molecule [222,223].

Monoclonal antibodies are immunoglobulins designed to bind specific antigens and represent the second most common form of targeted therapy [213,218]. Unlike small-molecule inhibitors, monoclonal antibodies are larger molecules that cannot enter cells. Instead, they work by targeting receptors on the surfaces of cancer cells, thereby blocking the molecules that signal proliferation or angiogenesis [214,224]. This approach is primarily used to target the antigens associated with oncogene signaling, thus inhibiting the pathways that promote cancer cell growth and survival [225]. The most common clinical applications of monoclonal antibodies are trastuzumab (targeting HER2), cetuximab (targeting EGFR), and pembrolizumab (targeting the PD-1/PD-L1 axis) [226,227,228]. Trastuzumab, for example, effectively disrupts oncogenic signaling by downregulating HER2, an RTK that is commonly overexpressed in *HER2*-positive breast cancer, thereby promoting its internalization and degradation [229,230]. Similarly, cetuximab and panitumumab bind to EGFR, preventing ligand binding and receptor dimerization, which inhibits oncogene signaling [231,232]. Moreover, the use of antibody therapy extends to tumor suppressors, as treatments with pembrolizumab or nivolumab have been shown to restore T-cell functionality against tumors, compensating for the LOF mutations in tumor suppressors [231,233].

Immunotherapy is the third major treatment method and a promising therapeutic approach, especially in people with oncogene-addicted cancers—cancers that depend heavily on a single oncogene or pathway [234]. This treatment increases the ability of the immune system to recognize and eliminate cancer cells, mainly through immune checkpoint inhibitors that block pathways like PD-1, PD-L1, and CTLA-4, which tumors exploit to decrease immune responses [235]. Notably, pembrolizumab and nivolumab, previously discussed as monoclonal antibodies, overlap with this category, as they release the brakes on T cells and are very effective against oncogene-driven cancers that have mutations in genes like *KRAS* and *EGFR* [236,237]. Tumors with mutated *KRAS* are likely to respond better due to upregulated PD-L1 expression and immune cell infiltration, as opposed to *EGFR* and *ALK*-driven tumors, which typically have “cold” tumor microenvironments with fewer immune cells [234,238,239]. Immunotherapy may also improve outcomes for tumors with genomic instability, including mutations in tumor-suppressor genes such as *TP53*, *STK11*, and *KEAP1* [237,240]. Moreover, immunotherapy has also shown success when combined with chemotherapy and anti-angiogenesis agents like bevacizumab, which work synergistically to change the tumor microenvironment [238,241].

Radioimmunotherapy (RIT) is an extension of immunotherapy that combines targeted radiation with monoclonal antibodies to selectively target and destroy cancer cells [242]. This approach delivers a high dose of therapeutic or tracer radiation while minimizing exposure to normal cells [243]. Recent studies have focused on optimizing the combination of targeted radiation and immunotherapy, particularly in treatments that use alpha (radium-223 and actinium-225) and beta radionuclides (90y-ibritumomab tiuxetan), which have shown cytotoxic effects in treating leukemia, prostate cancer, and non-Hodgkin lymphoma [244,245]. Beyond its cytotoxic capabilities, RIT can influence oncogene and tumor-suppressor gene activity. For instance, a study by Guo et al. [246] identifies the correlation between p53 and RIT efficacy in tumors with wild-type *TP53*. The study found that, in response to RIT, the activity of p53 was upregulated, which led to increased apoptosis and better regulation of DNA damage in cancer cells. This suggests that RIT could benefit patients with functional tumor-suppressor pathways, serving as an alternative therapy in cases resistant to conventional treatments [246].

Antibody–drug conjugates (ADCs) are a class of targeted cancer therapy that connects monoclonal antibodies with a potent cytotoxic drug (payload) through a chemical linker [247]. The monoclonal antibody offers a highly specific targeting capability, thus binding to a target antigen on the cancer cell’s surface. The presence of a chemical linker ensures that the payload, which has a highly potent cytotoxic effect, is released only inside the cancer cell, therefore minimizing the damage to healthy tissues [248]. The first FDA-approved ADC was the anti-CD33-targeted agent gemtuzumab ozogamicin in 2000, to treat patients with acute myeloid leukemia [249]. Since then, there have been eleven FDA-approved ADCs (Table 3) for targeting various tumor antigens, such as CD19, CD22, CD30, CD33, and CD79b in blood cancers (myeloma, lymphoma, and leukemia), and HER2, tissue factor, folate factor alpha, Nectin-4, and Trop-2 in solid cancers (NSCLC, breast cancer, gastric cancer, and ovarian cancer, among others [250], and many more are in advanced stages of clinical trials.

Chimeric antigen receptor (CAR) T-cell therapy is a novel cancer therapy that uses patients’ own T cells to fight cancer [251]. Usually, the T cells do not present receptors specific to the cancer cells’ antigens, which prevents them from attaching to the antigens and destroying the cancer cells. In CAR T-cell therapy, T cells are extracted from the patient’s blood and undergo genetic modification, which introduces a gene that encodes a cancer-specific antigen receptor on their cellular membrane, enabling them to recognize and attach to the cancer cell [252]. Then, CAR T cells are infused back into the patients, where they circulate and attack cancer cells. This therapy has shown a significantly greater promise in targeting and combating circulating blood cancers like leukemia, lymphomas, and myelomas compared to solid tumors, mostly because of the solid tumors’ inaccessibility due to their complex microenvironment [253]. So far, there are six FDA-approved CAR T-cell products for treating hematological malignancies (Table 4), and many more are in active clinical trials [254].

Despite the significant advancements and successes of targeted therapies and immune therapy, these approaches have many persistent limitations that hinder their success. Cancer cells frequently adapt, developing resistance that reduces the effectiveness of treatments like monoclonal antibodies, small-molecule inhibitors, ADCs, CAR T-cell products, and immune checkpoint inhibitors over time [255]. The resistance often stems from genetic mutations, altered signaling pathways, or changes in the tumor microenvironment, such as variable vasculature and immune suppression [256]. Additionally, these therapies can cause a spectrum of secondary effects that impact patients’ quality of life, including but not limited to skin toxicity, high blood pressure, and heart damage to severe autoimmune reactions like cytokine release syndrome (CRS), neurotoxicity, swelling, nausea, vomiting, diarrhea or constipation, allergic reactions, and hair loss [257]. Moreover, the complexity of these treatments, especially ADCs and CAR T-cell products, demands specialized manufacturing and delivery, increasing the costs and limiting their widespread availability and accessibility [258,259]. Addressing these challenges and limitations of targeted cancer therapies is crucial for improving therapeutic outcomes and treatment strategies to overcome these challenges.

### 4.3. Personalized Medicine Applications

Personalized medicine, also known as precision medicine, is a novel emerging approach that aims to determine disease patterns and develop devices and drugs tailored to individual patients [260]. This approach has been particularly intriguing in oncology, as it considers the inter- and intra-tumor variability in genetic profiles, tumor environments, and the oncogenic drivers specific to each tumor [261]. Personalized medicine aims to understand tumor biology and the development of resistance mechanisms, thus helping identify the tumor-specific biomarkers that factor into therapy-induced toxicities [261,262]. The majority of these techniques rely on NGS for cost- and time-effective tumor DNA sequencing to allow clinicians and researchers to better understand the unique genomic composition of each malignancy [263]. Previously discussed targeted therapies are at the forefront of emerging personalized medicine technologies [264]. However, personalized medicine includes other approaches like pharmacogenomics that not only focus on targeting oncogenic mutations but also aim to enhance drug metabolism and efficiency [265].

Pharmacogenomics offers a very compelling approach in the realm of personalized medicine through its implementation of genotyping technology to select and tailor drugs, predict drug delivery and dosage, anticipate adverse reactions, and ultimately improve treatment efficacy and prevention [265,266]. This field covers proteomics, transcriptomics, metabolomics, and metagenomics to develop single-gene drug therapies [267]. Rapalogs are currently the leading experimental candidates in advanced clinical trials that work by inhibiting the mTOR protein kinase. Notably, rapamycin and its analogs, temsirolimus, everolimus, and AP23573, are being tested and have generally shown good tolerance despite dose-dependent toxicity in some studies [91,268]. mTORC1 is sensitive to rapamycin, and the drug works by inhibiting downstream targets like 4E-BP1 and S6K1, which are involved in protein synthesis and cell growth, thus slowing down tumor progression [269]. However, rapamycin’s effect paradoxically leads to AKT activation by disrupting the mTOR/S6K-mediated feedback loop, which can unintentionally promote survival signals in cancer cells [90,91,268]. Despite the promising prospects of this approach, further research is needed to assess the drugs’ interference with metabolism and to address the resistance mechanisms that arise from the activation of the mTOR pathway [268,270]. Precision medicine still relies on co-administration with other therapies, involving the integration of NGS techniques, pharmacogenomics, and targeted therapies to minimize the adverse effects and setbacks in patient outcomes. The ongoing development and integration of emerging technologies such as artificial intelligence (AI) models in diagnosis, new cell-sequencing methods, and liquid biopsy techniques may open new avenues for providing comprehensive treatments for cancers that are driven by oncogenes and tumor-suppressor genes [260,271,272,273].

## 5. Emerging Technologies and Future Perspectives

### 5.1. Single-Cell Sequencing

Single-cell sequencing has emerged as a vital tool in analyzing tumor heterogeneity by offering the opportunity to sequence individual cells within a neoplastic lesion. The technique allows for the understanding of temporal heterogeneity and the evolution of cancer over time, showing the development of oncogenic mutations that lead to drug resistance. This technology allows for not only genomic, epigenomic, and transcriptional analysis but also offers insights into single-nucleotide variations, copy number variations, and structural variations within tumors [274,275,276]. The most widely used method of single-cell sequencing, single-cell RNA sequencing (scRNA-seq), begins with the isolation of a single cell from a tumor lesion using laser capture microdissection, where a laser precisely extracts individual cells from the tissue [277,278]. Cells are then kept in a lysis buffer and sorted into microtiter plates using fluorescence-activated cell sorting (FACS) [279]. The process of quickly and accurately isolating single cells without compromising their integrity is the biggest challenge for single-cell sequencing. For instance, using trypsin or collagenase in the buffer can impair cell viability and change the transcriptional profile. Following isolation, cells are treated with reverse transcriptase alongside a poly(t) primer, resulting in the creation of complementary DNA (cDNA), which is then amplified using a polymerase chain reaction or in vitro transcription [279,280,281,282]. Finally, sequencing is achieved using a sequencing platform such as Illumina.

Investigating tumor heterogeneity using single-cell sequencing offers valuable insight into the expression of oncogenes underlying drug resistance mechanisms. Using single-cell transcriptome sequencing, A study by Zhao et al. [283] utilized scRNA-seq to uncover increased tumor heterogeneity in a patient with recurring diffuse large B-cell lymphoma (DLBCL). The study identified malignant B-cell groups with upregulated genes coding for proteins involved in numerous oncogenic pathways, including *MYC*, *BCL2*, and *E2F* targets [283]. Zhao et al. [283] isolated two distinct malignant B-cell subgroups from a singular tumor, one of which revealed co-expression of *MYC* and *BCL2*. The co-expression of *MYC* and *BCL2* is a hallmark of double-expression lymphoma (DEL) and is linked to poor overall prognosis in patients [284,285]. Further analysis by Zhao et al. [283] showed that cell populations with a double expression of *MYC*/*BCL2* exhibited upregulation of inflammatory and immune-related signaling pathways, which were previously shown to promote tumor invasion and metastasis [286]. Meanwhile, the second isolated cell population of malignant B-cells lacked the double expression of *MYC*/*BCL2*, therefore lacking the pro-inflammatory capabilities [283]. However, this population showed a marked reduction in MHC Class I and II expression [283]. Low MHC expression in tumor cells results in low immunogenicity and immune evasion capabilities, which also leads to a poor patient outcome [287]. scRNA-seq of this tumor revealed the heterogeneous nature of the present cells, where despite differing in *MYC*/*BCL2* expression, each malignant B-cell population contained adaptations that would promote multi-drug resistance and tumorigenesis, particularly in diffuse large B-cell lymphoma (DLBCL) [283]. A similar study employed scRNA-seq to monitor the expression of the KIT F522C mutation in mast cell leukemia before and after 10 months of treatment with midostaurin, a chemotherapeutic agent that inhibits KIT signaling [288]. The *KIT* gene encodes a receptor tyrosine kinase involved in cell proliferation and migration, which gains oncogenic function when mutated or upregulated in cancer cells [289]. Pathogenic mutation in *KIT* is found in 80% of systemic mastocytosis cases, including mast cell leukemia [290]. Single sequencing of peripheral blood after treatment with midostaurin showed a decrease in cells expressing a *KIT* GOF mutation. However, an increase in the cells exhibiting a mutation in the runt-related transcription factor 1 gene (*RUNX1*) was also observed. *RUNX1* acts as an oncogene by enhancing the Wnt/β-catenin signaling pathway, promoting metastasis and poor prognosis [291,292].

Multi-omics is an integrative approach that combines multiple layers of biological data, such as genomics, transcriptomics, proteomics, metabolomics, etc. (Table 5) [293].

The term “omics” refers to a comprehensive quantitative analysis of molecules in a sample to understand its underlying molecular mechanisms [294]. This approach is especially powerful in oncogene and tumor-suppressor research when combined with single-cell sequencing, as it allows researchers to observe molecular changes at a single-cell resolution [295]. The availability of a multi-omics approach represents a new era in cancer therapy. It uses algorithmic frameworks to identify crucial genomic alterations, making it a necessary tool for tumor classification, diagnostics, and prognostication [296]. One of the key applications of multi-omics is its role in the classification of tumor-suppressor genes [297]. Large-scale initiatives of multi-omics data, such as The Cancer Genome Atlas (TCGA) datasets, have made identifying tumor suppressors and other biomarkers in various cancers much more convenient [298,299]. By utilizing transcriptomics and functional genomic data to analyze breast cancer, one study revealed the significant role of the ribosome pathway in gene regulation of tumor suppressors. The study also highlighted that certain processes involved in mRNA processing are more active and are connected to key functions linked with angiogenesis and proliferation, which are very important for tumor development [297]. Furthermore, multi-omics can enhance the analysis of intratumoral heterogeneity when combined with NGS techniques, which help reveal the nuclear aberrations in tumors [300]. Multi-omics produces an extensive quantity of data that can be difficult to interpret. Maximizing the full potential of the high-throughput data from different molecular layers forces high costs and demands for more sophisticated analytical technologies [301].

### 5.2. CRISPR-Cas9 Screening

Recent advances in gene-editing technologies have significantly improved our understanding of the roles of oncogenes and tumor-suppressor genes in cancer pathology. The Clustered Regularly Interspaced Short Palindromic Repeats (CRISPR)-associated endonuclease 9 (Cas9) protein has been developed as a powerful tool to rapidly and precisely induce oncogenic mutations within a cell population [302]. The Cas9 protein functions as an endonuclease that cleaves DNA to produce double-stranded breaks or single-strand nicks, creating new genetic sequences to study the resulting phenotypic changes [303]. While previous technologies, such as zinc-finger nucleases and transcription activator-like effector nucleases, used specific customizable proteins that offered advantages in gene editing, they were difficult to apply across whole-genome investigations.

CRISPR-Cas9 screening begins with recognizing a target gene sequence by using a single guide RNA (sgRNA), which then activates the Cas9 endonuclease to create a double-stranded break [304]. Following the introduction of the desired sequence, the double-stranded break is then repaired using the cell’s native non-homologous end joining or homology-directed repair system, depending on the cell-cycle stage in which the break was introduced [304]. Variations of Cas9 have been developed to improve its endonuclease activity. For example, the Cas9 mutant nickase (Cas9n) has been developed to introduce more site-specific double-stranded breaks while retaining the efficiency of the wild-type Cas9 protein [305]. By targeting specific gene sequences and observing phenotypic effects, vast libraries of sgRNA targets have been compiled to screen for essential genes in cancer and those associated with drug resistance [302].

Therapeutic targeting of oncogenes and tumor-suppressor genes using CRISPR-Cas9 technology has successfully identified their roles in affecting downstream mechanisms. One recent study used CRISPR-Cas9 to introduce a mutation in the *BRAF* gene in stem cells carrying a fusion mutation characteristic of angiomatoid fibrous histiocytoma (AFH) [306]. AFH tumors are known to be driven by the oncogenic fusion of the EWS RNA-binding protein 1 (EWSR1) and cAMP-responsive element-binding protein 1 (CREB1), making them a primary focus of targeted therapies [307]. By using CRISPR-Cas9 to induce *BRAF* c.1799 T > A point mutation in human embryonic stem cells with the *EWSR1*/*CREB1* fusion, Vanoli et al. [306] confirmed an upregulation of the downstream pathways that facilitate cancer proliferation. Upregulated genes included protein tyrosine phosphatase receptor type N (*PTPRN*), which is associated with cell growth, mitosis, and oncogenic transformation, as well as tissue-type plasminogen activator (*PLAT*), versican (*VCAN*), and transforming growth factor beta 1 (*TGF-β1*), which play roles in tumor cell migration, adhesion, and proliferation [306]. CRISPR-Cas9 methods were vital in elucidating the role of *BRAF* in promoting the proliferation of AFH, as these effects were not observed in human stem cells that exhibited only the *EWSR1*/*CREB1* fusion [306].

Beyond manipulating and analyzing tumor cell samples, CRISPR-Cas9 holds potential for somatic gene therapy, aiming to introduce new genetic material in vivo to make the cancer more susceptible to treatment [308]. Current studies show limitations in achieving clinical success due to gene silencing, host immune response, and off-target effects [309]. However, nanotechnologies are being developed to reduce some of these adverse effects by using nanocarriers to deliver CRISPR-Cas9 components into cells, offering the potential for in vivo gene therapy in the future [310].

### 5.3. Artificial Intelligence and Machine-Learning Applications

Artificial intelligence (AI) has quickly become an emerging technology that has transformed the personalized approach to cancer medicine, including screening, diagnosis, early detection, and treatment [260]. For example, AI classification systems have been shown to be successful in differentiating between carcinoma and non-lactating metastasis in breast cancer, with the convolutional neural network (CNN) being particularly effective in detecting breast cancer with high accuracy [273]. AI technologies rely on algorithms such as machine learning (ML) and deep learning to analyze patients’ clinical variables and medical data, assisting in the treatment of lung cancer cases [311]. The diagnosis of lung cancer relies on early screening for lung nodules, which have a high likelihood of malignancy if larger than 3 cm [312,313]. Computer-aided diagnoses (CAD) tools have been developed to work with lung cancer databases like the Lung Image Database Consortium (LIDC) and the Early Lung Cancer Action Program (ELCAP) to diagnose malignancy in lung nodules [314,315,316]. In one study, 6400 images of 978 nodules were analyzed using the CAD system, achieving an accuracy, sensitivity, and specificity of 93.2%, 92.4%, and 94.8%, respectively, for malignancy diagnosis [317]. Studies have demonstrated that AI can also predict oncogenic mutations in lesions based on the morphological characteristics of NSCLC in hematoxylin–eosin-stained images [311]. Genetic alterations in the epidermal growth factor receptor (EFGR) and anaplastic lymphoma kinase (ALK) have been used as valuable predictive markers in NSCLC, with *EFGR* mutation prediction playing a vital role in selecting treatment [318]. AI analysis has been successful in predicting the mutations present in six common oncogenes (*STK11*, *EGFR*, *FAT1*, *SETBP1*, *KRAS*, and *TP53*), offering an accurate and cost-effective method for identifying the genetic markers that are important for treatment planning [319].

ML is another important tool in the genomic analysis of cancers, which opens new opportunities for personalized medicine. For instance, molecular signatures from gene expression profiling have been analyzed in prostate cancers and applied to clinical decision-making in treatment [320]. One study used ML models to identify 30 genes with downregulation and hypermethylation at their promoter region, which were used to predict metastasis in patients with prostate cancer [321]. Additionally, the ML model identified five significantly mutated genes in patients with metastasis, which included *POLR3K* (RNA polymerase III subunit K), *EEF1D* (eukaryotic translation elongation factor 1 delta), *IGFALS* (insulin-like growth factor-binding protein acid labile subunit), *H2AW* (H2A.W histone), and *FASTK* (Fas-activated serine/threonine kinase) [321]. Developments in AI offer many exciting opportunities for accurate, noninvasive diagnosis and a deeper understanding of the expression of oncogenes and tumor-suppressor genes underlying the pathology of cancer.

### 5.4. Liquid Biopsy

**Figure 4:**
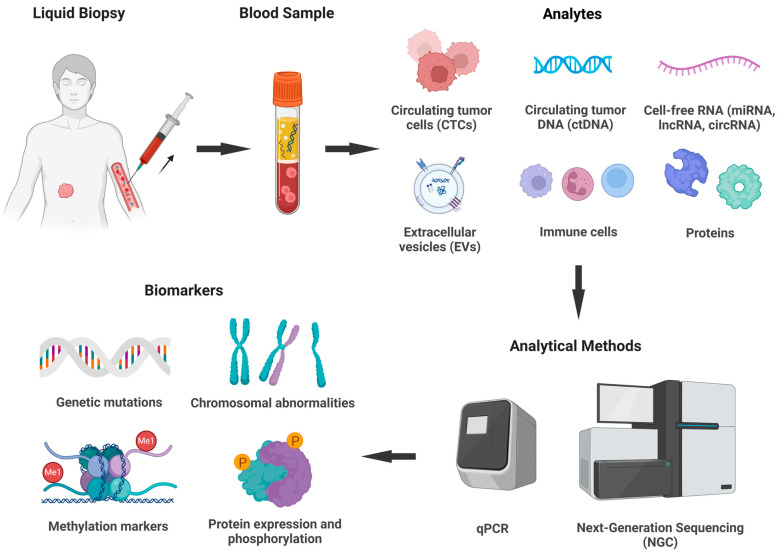
Liquid biopsy of bodily fluids (blood). Analytes collected through liquid biopsy include circulating tumor cells (CTCs), circulating tumor DNA (ctDNA), cell-free RNA (e.g., miRNA, lncRNA, circRNA), extracellular vesicles (EVs), immune cells, and proteins, which are then analyzed using advanced detection techniques such as PCR and next-generation sequencing (NGS) to identify specific cancer markers, even at low abundance. Liquid biopsy enables the discovery of various cancer-related biomarkers, including various genetic mutations, chromosomal abnormalities, DNA methylation patterns, tumor-associated protein expression, and post-translational modifications, such as phosphorylation. Created in BioRender. Stojchevski, R. (2025) https://BioRender.com/h75n317.

Tissue biopsies are a standard method for detecting and profiling tumors. While reliable, many limitations demand less invasive and more accurate approaches [322]. Unlike the surgical excision of malignant tissue samples, the analysis of cancer-related signals in biological fluids, known as liquid biopsy, has emerged as an alternative diagnostic tool [323]. The term “liquid biopsy” refers to the analysis of bodily fluids, such as blood, urine, cerebrospinal fluid, and saliva, to detect cancer-related biomarkers (Figure 4). Analytes collected from these fluids include circulating tumor cells (CTCs), circulating tumor DNA (ctDNA), cell-free RNA (miRNA, lncRNA, and circRNA), extracellular vesicles (EVs), immune cells, and proteins [271,322]. By analyzing these analytes, liquid biopsies can uncover genetic mutations, chromosomal abnormalities, DNA methylation patterns, tumor-associated protein expressions, immune cell activity profiles, etc. The method relies on advanced detection techniques such as PCR, NGS, and immunoassays to identify traditional and specific markers that are in low abundance in bodily fluids, making it especially useful in early screening, unlike traditional biopsies that occur at later stages of tumor development [322]. PCR tests with primers targeting tumor-specific transcriptions, mutations, translocations, and methylation patterns can detect CTCs, even in cases of low mRNA expression [324,325]. Additionally, NGS techniques like WES provide a more comprehensive cancer-gene panel, allowing for the characterization of ctDNA and exosomes, addressing the limitations of traditional laboratory techniques [322,326].

Liquid biopsies cover a broad range of clinical applications, including assessing immunotherapy response at checkpoint blockades, prognostication, early cancer detection, evaluating residual disease after treatment, early evaluation of response and resistance, and understanding tumor heterogeneity [327,328,329,330,331]. The diagnostic methodologies within liquid biopsy have shown promise in the isolation of oncogene mutations. Siravegna et al. [332] identified alterations in *KRAS*, *NRAS*, *MET*, *ERBB2*, *FLT3*, *EGFR*, and *MAP2K1* by studying patients’ ctDNA, highlighting their role in oncogenic signaling pathways. Moreover, using liquid biopsy, alterations in *BRAF*, *HER2*, *AKT*, and *ROS1* can be identified, proving it to be a useful way for studying oncogene-addicted cancers [333,334]. Beyond primary diagnosis, liquid biopsies can be employed throughout a patient’s treatment course to measure the progression and responsiveness of the treatment [335]. Minimal residual disease (MRD) refers to cancer cells persisting at undetectable levels in a patient after the treatment, posing a risk for recurrence [336,337]. The application of liquid biopsy analyses can identify very low concentrations of CTCs and ctDNA in blood samples, thus enabling MRD detection in patients with various malignancies [335,336,337]. However, it is important to note that liquid biopsies are still majorly limited due to their lack of sensitivity and precision in identifying tumor types to the same degree as tissue biopsy. These limitations also raise concerns about whether liquid biopsy samples are representative of all genomic clones within a tumor or just a specific sub-region [338,339].

### 5.5. Tumor Microenvironment, Cancer Vaccines, and Oncolytic Viruses

The tumor microenvironment (TME) is a complex network of immune cells, stromal cells, blood vessels, extracellular matrix (ECM), and signaling molecules that interact with the tumor cells [340]. The effectiveness of antitumor immunity is determined by the collaboration between innate and adaptive immune cells within the TME [341]. In this environment, natural killer (NK) cells, neutrophils, and macrophages drive rapid tumoricidal activity, while dendritic cells (DCs) present and process antigens for the activation of CD4^+^ and CD8^+^ T cells [341,342]. Once activated, T cells migrate to the TME, where they inhibit tumor cell proliferation and induce cell death via interferon γ (IFNγ), interleukin 2 (IL-2), and tumor necrosis factor (TNF)-dependent mechanisms [341]. Cancer-associated fibroblasts (CAFs) are a type of cell found in the TME that is activated in response to cancer, contributing to the overproduction of ECM, growth factors (VEGF), and cytokines (TGF-B), which promote tumor progression [343,344]. CAFs, along with tumor-associated macrophages (TAMs), have been shown to contribute to the extrinsic immunoresistance of cancers [341]. Recent research suggests that using MMP inhibitors or collagen crosslinking inhibitors can disrupt the formation of ECM by CAFSs, facilitating the migration of T cells and, thus, reinforcing the effect of immunotherapies [341,345].

**Figure 5:**
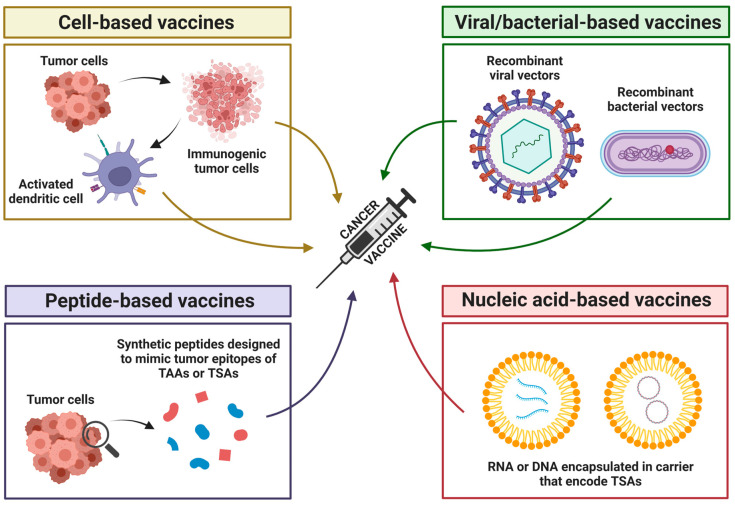
Types of cancer vaccines. There are four types of cancer vaccines: cell-based, viral/bacterial-based, peptide-based, and nucleic acid-based vaccines. Cell-based vaccines are prepared using whole tumor cells or tumor cell fragments. which can be injected directly or loaded onto dendritic cells along with adjuvants to enhance their immunogenicity and stimulate a stronger anti-tumor immune response. Viral/bacterial-based vaccines are designed using recombinant viral or bacterial vectors to deliver genetic material encoding cancer-specific proteins or antigens. These vectors infect host cells, enabling the expression of the target antigens and stimulating an immune response against cancer cells. Peptide-based vaccines use short biosynthetic peptides that mimic specific tumor epitopes of tumor-associated antigens (TAAs) or tumor-specific antigens (TSAs) to stimulate the immune system to recognize and attack cancer cells at specific tumor sites where the target antigens are expressed. Nucleic acid-based vaccines deliver genetic material (RNA or DNA) that encodes tumor-specific antigens. The RNA or DNA is typically encapsulated in carriers to protect it from degradation and facilitate efficient delivery into the host cells. Once inside, the genetic material is expressed, producing the target antigens, which are then presented to the immune system. This stimulates T and B cells to recognize and attack cancer cells that express these antigens. Created in BioRender. Stojchevski, R. (2025) https://BioRender.com/c04n703.

TME-associated cancer vaccination therapies are a novel extension of immunotherapy that offers revolutionary potential in oncology [346]. Cancer vaccines can be clinically used therapeutically or preventively and are delivered in four forms: cell-based, viral/bacterial-based, peptide-based, and nucleic acid-based vaccines (Figure 5) [347,348]. These vaccines use tumor-associated antigens (TAAs) and tumor-specific antigens (TSAs) to elicit an immune response in patients that would provoke both cellular and humoral immune responses to eradicate tumors and prevent tumorigenesis [348,349]. Cell-based vaccines are prepared using whole tumor cells or cell fragments, which can be injected directly or loaded on DCs with adjuvants to enhance immunogenicity [348,350]. Viral/bacterial-based vaccines are naturally immunogenic, and their genetic material can be engineered to express tumor antigens [348]. Peptide-based vaccines contain biosynthetic peptides that represent known tumor antigens to stimulate the immune system to attack particular tumor sites [348]. Lastly, nucleic acid-based vaccines deliver genetic material that encodes tumor antigens, thus inducing MHC I-mediated CD8^+^ T-cell responses, making it one of the more promising approaches [348,351].

Therapeutic cancer vaccines have shown great success in clinical trials [347]. Several therapeutic vaccines that have been approved by the FDA are already in use against various cancers (Table 6) [347].

Sipuleucel-T was the first FDA-approved therapeutic cell-based vaccine for metastatic prostate cancer [352]. The prolonged disease course of advanced prostate cancer creates a window where the body can generate an immune response against the cancer cells [353]. Another example is the bacillus Calmette–Guerin (BCG) vaccine, which is a bacterial-based vaccine used to treat early-stage bladder cancer [347]. BCG uses inactivated tuberculosis bacteria, which is administered through a catheter to stimulate an immune response, causing apoptosis, necrocytosis, and oxidative stress [354,355]. Despite recent successes, therapeutic cancer vaccines face several challenges, such as difficulties in finding cancer-specific antigens, as some antigens can be present on both normal and cancerous cells, which could cause unwanted side effects, or the production of molecules by the cancer cells that suppress the immune response [347].

While therapeutic vaccines target existing tumors, prophylactic/preventive cancer vaccines aim to reduce the initial risk of cancer development, primarily protecting against virus-induced cancers [347]. One of the two currently approved and common prophylactic cancer vaccines is the Human Papillomavirus (HPV) vaccine (Table 6), which utilizes a virus-like particle of the non-oncogenic and non-infectious papillomavirus capsid protein L1 to build an immune response that would prevent HPV from inserting itself into the host’s genome and cause nuclear aberrations [356,357]. The other prophylactic vaccine in use is the Hepatitis B vaccine, which is a common liver infection that leads to liver cirrhosis and hepatocellular carcinoma (HCC) (Table 6) [358]. A study by Cao et al. [359] showed that the Hepatitis B vaccine offers 72% protection against liver cancer post-infection.

Oncolytic viruses (OVs) are a novel immunotherapy that functions similarly to viral-based cancer vaccines to specifically target and kill tumor cells and promote anti-tumor immune responses [348]. The OV infects the tumor cells, inducing ROS production and cytokine release to stimulate immune cells and subsequently release TAAs [348,360]. OVs can induce various forms of immunogenic cell death, such as apoptosis, necroptosis, and pyroptosis, stimulating the dying host cells to release damage-associated molecular patterns (DAMPs), creating a pro-inflammatory environment that advances the maturation of antigen-presenting cells to further activate the immune response [361,362,363,364]. Moreover, OVs can be synergistically used with immune checkpoint inhibitors, such as anti-PD-1/PD-L1 and anti-CTLA-4 antibodies, to modulate the TME and strengthen the immune response [364,365]. Some OVs that are considered possible vehicles for oncoviral therapy are the herpes simplex virus, measles, mumps, adenovirus, retrovirus, and parvovirus, among others [366]. Talimogene laherparepvec (T-VEC), the first approved OV immunotherapy, treats metastatic melanoma by successfully activating tumor-specific effector T cells and TAAs (Table 6) [367]. Adenoviruses and herpes simplex viruses are the most promising OVs, as they are easy to manipulate, have a clear genetic structure, can easily achieve gene transfer and tumor antigen expression, have a broad spectrum of host cell tropism, and can be prepared in large quantities [368,369,370].

Cancer vaccines and OVs hold promise as emerging cancer therapies, but they face significant limitations. Cancer vaccines often struggle with weak immunogenicity and tumor heterogeneity, reducing their effectiveness [346,371]. Similarly, oncolytic viruses are limited by delivery challenges, immune clearance, and variable tumor susceptibility [372]. These hurdles highlight the need for further research to optimize their clinical impact.

## 6. Conclusions

The interaction between tumor-suppressor genes and oncogenes represents an essential area of research for enhancing our understanding of genomic treatments for cancer. By clarifying the roles of these genes in regulating the key signaling pathways involved in cell proliferation—such as p53, Rb, Ras/Raf/ERK/MAPK, PI3K/AKT, and Wnt/β-catenin—clinicians and researchers will be better equipped to develop therapeutic strategies that target specific genomic alterations in patients. As studies reveal how these genes interact with one another and fulfill various tumorigenic roles, they provide a more comprehensive perspective on the complexities of tumor heterogeneity due to multiple genetic alterations. This understanding is further improved by exploring the tumor microenvironment, where innate and adaptive immune cells (NK cells and DCs) promote antitumor responses. Most importantly, as recent research progresses, these insights will support the development of novel technologies for sequencing, diagnosing, and treating cancer at a genomic level in a personalized manner, thereby potentially revolutionizing the field of oncology and genomics.

Advancements in sequencing technologies, such as NGS methods and, particularly, single-cell sequencing methods, stand out as the most significant developments in genomics and have significant implications in diagnostics, preventative screening, and progression monitoring. By focusing on short- and long-coverage genome sequencing, these emerging techniques can efficiently identify a broad spectrum of genetic alterations, ranging from SNVs, CNVs, insertions, and deletions. These methods can spot existing mutations and discover genomic regions that are susceptible to mutation. NGS unlocks the potential for less-invasive procedures, such as liquid biopsy, that can monitor tumorigenesis more efficiently than traditional methods by detecting and sequencing cancer biomarkers, establishing transcriptional profiles, evaluating residual disease after treatment, and predicting and identifying morphological variations between tumors. While liquid biopsy offers depth and quantity in identifying tumorigenic manifestations, it still lacks the sensitivity and precision of traditional tissue biopsy. Additionally, the sequencing techniques generate a large amount of data for laboratories to process and interpret, which calls for advancements in bioinformatics to improve analytical efficiency. The integration of AI and ML into oncology can overcome these limitations to improve the existing and develop new diagnostic approaches. Using advanced algorithms, AI models can identify the predictive markers and epigenetic changes of tumor suppressors and oncogenes and differentiate between the pathology and morphology associated with tumor heterogeneity. Moreover, CRISPR-Cas9 offers a revolutionary tool for directly manipulating the specific gene sequences that are responsible for tumorigenesis and drug resistance mechanisms, further improving the precision of genomic interventions. Finally, the insights into the tumor microenvironment enable innovations in cancer vaccines and oncolytic virus therapies by enhancing the ability of the immune system to detect and target tumor cells, thus edging closer to truly personalized and effective prevention, detection, diagnosis, and treatment of cancer.

## Figures and Tables

**Table 1:** Examples of genes with dual oncogenic and tumor-suppressive roles.

Gene	Primary Role	Dual Role	Condition	Biological Mechanism
*TP53*	Tumor Suppressor	Oncogene	GOF mutations (e.g., R175, R248)	Altered transcriptional activity; activating pro-oncogenic genes (e.g., growth factors)
*MYC*	Oncogene	Tumor Suppressor	Overexpression	MDM2-p53 apoptotic stress response; upregulating pro-apoptotic targets (e.g., BAX)
*TGFβ1*	Tumor Suppressor	Oncogene	Pathway defects (e.g., SMAD loss)	Signaling cascade activation; enhancing angiogenesis via VEGF and immune evasion

**Table 2:** FDA-approved small-molecule inhibitors in use for treatment of various cancers.

Inhibitor	Target	Mechanism of Action	FDA Approval
Erlotinib	EGFR (RTK)	Competitively inhibits ATP binding to EGFR; Blocks downstream signaling	2004 (NSCLC), 2005 (Pancreatic cancer)
Gefitinib	EGFR (RTK)	Inhibits EGFR tyrosine kinase activity; reduces cell proliferation	2003 (NSCLC)
Lapatinib	HER2, EGFR (RTKs)	Dual inhibitor; prevents phosphorylation and signaling	2007 (HER+ Breast cancer)
Sunitinib	VEGFR, PDGFR, KIT (RTKs)	Inhibits multiple RTKs; leads to antiangiogenic and antiproliferative effects	2006 (pNET, RCC, GIST)
Sorafenib	VEGFR, PDGFR, KIT (RTKs)	Inhibits RTKs and RAF kinase; blocks angiogenesis and tumorigenesis	2005 (HCC, RCC, Thyroid cancer)
Vemurafenib	BRAF V600E (MAPK Pathway)	Selectively inhibits mutant BRAF; prevents aberrant MAPK activation	2011 (Melanoma, Erdheim–Chester disease)
Dabrafenib	BRAF V600E (MAPK Pathway)	Inhibits mutant BRAF kinase; reduces MAPK-driven cell proliferation	2013 (Melanoma, NSCLC, Anaplastic thyroid cancer)
Encorafenib	BRAF V600E (MAPK Pathway)	BRAF kinase inhibitor; blocks mutant BRAF kinase; inhibits MAPK pathway signaling.	2018 (Melanoma), 2020 (Colorectal cancer), 2023 (NSCLC)
Binimetinib	MEK1/2 (MAPK Pathway)	Blocks MEK1/2 activity; inhibits downstream MAPK pathway signaling.	2018 (Melanoma), 2023 (NSCLC)
Trametinib	MEK1/2 (MAPK Pathway)	Inhibits MEK1/2; blocks MAPK activation downstream of BRAF	2013 (Melanoma, NSCLC, Anaplastic thyroid cancer)
Cobimetinib	MEK1/2 (MAPK Pathway)	Selectively inhibits MEK; suppresses MAPK signaling	2015 (Melanoma)

**Table 3:** FDA-approved antibody–drug conjugates (ADCs) for treatment of various cancers.

ADC Generic Name	Target Antigen	Cytotoxic Payload	FDA Approval
Loncastuximab tesirine	CD19	SG3199, alkylating agent (DNA targeting)	2021 (Diffuse Large B-Cell Lymphoma—DLBCL)
Inotuzumab ozogamicin	CD22	Calicheamicin (cytotoxic antibiotic)	2017 (B-cell Acute Lymphoblastic Leukemia—ALL)
Brentuximab vedotin	CD30	Monomethyl auristatin E (microtubule targeting)	2011, 2015, 2018 (Hodgkin lymphoma—HL; 2011, 2017, 2018 (Anaplastic Large Cell Lymphoma—ALCL); 2018 (Peripheral T-Cell Lymphoma—PTCL)
Gemtuzumab ozogamicin	CD33	Calicheamicin (cytotoxic antibiotic)	2017 (Acute Myeloid Leukemia—AML)
Polatuzumab vedotin	CD79b	Monomethyl auristatin E (microtubule targeting)	2019, 2023 (DLBCL)
Trastuzumab emtansine	HER2	DM1 (microtubule targeting)	2013, 2019 (HER2+ Breast Cancer)
Trastuzumab deruxtecan	HER2	Topoisomerase I inhibitor (DNA targeting)	2019, 2022 (HER2+ Breast Cancer); 2021 (Gastric Adenocarcinoma—GAC or Gastroesophageal Junction—GEJ Adenocarcinoma); 2022 (NSCLC)
Tisotumab vedotin	Tissue Factor	Monomethyl auristatin E (microtubule targeting)	2021 (Cervical Cancer)
Mirvetuximab soravtansine–gynx	Folate Receptor Alpha	DM4 (microtubule targeting)	2022 (Ovarian Cancer, Fallopian Tube Cancer, and Peritoneal Cancer)
Enfortumab vedotin	Nectin-4	Monomethyl auristatin E (microtubule targeting)	2019, 2023 (Urothelial Cancer)
Sacituzumab govitecan	Trop-2	SN-38 topoisomerase-1 inhibitor (DNA targeting)	2020 (Triple-Negative Breast Cancer—TNBC); 2021 (Urothelial Cancer); 2023 (HER2- Breast Cancer, HR+ Breast Cancer)

**Table 4:** FDA-approved CAR T-cell products for treatment of hematological malignancies.

CAR T-Cell Product Generic Name	Target Antigen	FDA Approval
Tisagenlecleucel	CD19	2017 (ALL); 2018 (DLBCL); 2022 (Follicular lymphoma—FL)
Axicabtagene ciloleucel	CD19	2017, 2022 (DLBCL, PMBCL); 2021 (FL)
Brexucabtagene autoleucel	CD19	2020 (Mantle Cell Lymphoma—MCL); 2021 (ALL)
Lisocabtagene maraleucel	CD19	2021, 2022, 2024 (DLBCL, PMBCL)
Idecabtagene vicleucel	BCMA	2021, 2024 (Multiple Myeloma—MM)
Ciltacabtagene autoleucel	BCMA	2022, 2023 (MM)

**Table 5:** Different levels of omics and their analytical techniques.

Omics Level	Description	Analytical Techniques
Genome	Study of the complete genome of organisms	NGS, WGS, Sanger Sequencing, SNP Sequencing
Transcriptome	Study of the messenger RNA transcripts and gene expression	Northern Blotting, Serial Analysis of Gene Expression, RNA Sequencing, DNA Microarrays
Proteome	Study of protein levels present in the organism	Mass Spectrometry, SDS-PAGE, Multidimensional Protein Identification Technology
Metabolome	Study of small molecules (i.e., amino acids, sugars, and fatty acids) that are metabolized	Mass Spectrometry, Nuclear Magnetic Resonance
Interactome	Study of protein–protein interactions in signal transduction, transcriptional regulation, and metabolic pathways	Mass Spectrometry, Tandem Affinity Purification, Two-Hybrid System

**Table 6:** FDA-approved cancer vaccines.

Vaccine Name	Type	Key Details	Prophylactic/ Therapeutics	FDA Approval
Sipuleucel-T (Provenge)	Cell based	Autologous dendritic cells activated with PAP-GM-CSF fusion protein	Therapeutic	2010 (Metastatic castration- resistant prostate cancer mCRPC)
Bacillus Calmette-Guerin (BCG)	Bacterial based	Live attenuated bacterium; stimulates immune response against bladder tumors	Therapeutic	1990 (Non-muscle-invasive bladder cancer NMIBC)
Talimogene Laherparepvec (T-VEC, Imlygic)	Viral based (Oncolytic)	Modified herpes virus; lyses tumors and enhances antitumor immunity	Therapeutic	2015 (Melanoma)
Hepatitis B (HBV) Vaccine (Recombivax HB, Energix-B)	Viral based (Recombinant protein)	Prevents HBV infection, indirectly reducing HCC	Prophylactic	1986 Recombivax HB 1989 Energix-B, (Hepatitis B virus—prevents HCC)
HPV Vaccines (Cervarix, Gardasil 9)	Viral based (Virus replicon particle)	Targets HPV strains (i.e., 16/18) directly linked to HPV-related cancer and prevents infection	Prophylactic	2009 Cervarix, 2014 Gardasil 9 (HPV-related cancers— prevents cervical, anal, and other types of cancers)

## Data Availability

No new data were created.

## Abbreviations

The following abbreviations are used in this manuscript:

ABC	ATP-Binding Cassette
ADC	Antibody–Drug Conjugate
AFH	Angiomatoid Fibrous Histiocytoma
AI	Artificial Intelligence
AKT	Protein Kinase B
ALL	Acute Lymphoblastic Leukemia
ALK	Anaplastic Lymphoma Kinase
ALV	Avian Leukosis Virus
AML	Acute Myeloid Leukemia
APC	Adenomatous Polyposis Coli
AS-PCR	Allele-Specific Polymerase Chain Reaction
BAD	Bcl-2-Associated Death Promoter
BCG	Bacillus Calmette–Guerin
BCRP	Breast Cancer Resistance Protein
Bcl-2	B-cell Lymphoma-2
bp	Base Pairs
BRCA1	Breast Cancer Gene 1
CAF	Cancer-Associated Fibroblasts
CAD	Computer-Aided Diagnoses
CAR	Chimeric Antigen Receptor
Cas9	CRISPR-associated Endonuclease 9
Cas9n	Cas9 Mutant Nickase
CCNE	Cyclin E
CDK	Cyclin-Dependent Kinase
CDKN2A	Cyclin-Dependent Kinase Inhibitor 2A
cDNA	Complementary DNA
c-MYC	Cellular Myelocytomatosis Oncogene
CKI	Cyclin-Dependent Kinase Inhibitors
CNN	Convolutional Neural Network
CML	Chronic Myelogenous Leukemia
CNV	Copy Number Variations
CRS	Cytokine Release Syndrome
CRISPR	Clustered Regularly Interspaced Short Palindromic Repeats
CTNNB1	Catenin-Beta-1
CTCs	Circulating Tumor Cells
ctDNA	Circulating Tumor DNA
DAMP	Damage-Associated Molecular Patterns
DC	Dendritic Cell
DEL	Double-Expression Lymphoma
DLBCL	Large B-cell Lymphoma
DNMT	DNA Methyltransferase
DVL	Disheveled Protein
ECM	Extracellular Matrix
EGF	Epidermal Growth Factor
EFGR	Epidermal Growth Factor
ELCAP	Early Lung Cancer Action Program
EMT	Epithelial to Mesenchymal Transition
ERK	Extracellular Signal-Regulated Kinase
EWSR1	EWS RNA-Binding Protein 1
EVs	Extracellular Vesicles
FACS	Fluorescence-Activated Cell Sorting
FASTK	Fas-Activated Serine/Threonine Kinase
FDA	U.S. Food and Drug Administration
FFPE	Formalin-Fixed Paraffin Embedded
FL	Follicular Lymphoma
FOXO	Forkhead Box O
GAC	Gastric Adenocarcinoma
GADD45	Growth Arrest and DNA Damage-Inducible 45
GEF	Guanine Nucleotide Exchange Factors
GEJ	Gastroesophageal Junction Adenocarcinoma
GIST	Gastrointestinal Stromal Tumor
GOF	Gain of Function
GSK-3β	Glycogen Synthase Kinase-3β
H2AW	H2A.W Histone
H3K27me3	Trimethylation of Histone H3 at Lysine 27
HCC	Hepatocellular Carcinoma
HER2	Human Epidermal Growth Factor Receptor 2
HL	Hodgkin Lymphoma
HPV	Human Papillomavirus
HR+	Hormone Receptor Positive (Estrogen and Progesterone)
IARC	International Agency for Research on Cancer
EEF1D	Eukaryotic Translation Elongation Factor 1 Delta
IFNγ	Interferon gamma
IGF-1	Insulin-Like Growth Factor-1
IGFALS	Insulin-Like Growth Factor Binding Protein Acid Labile Subunit
IL-2	Interleukin 2
kbp	Kilobase Pairs
LIDC	Lung Image Database Consortium
LOF	Loss of Function
LOH	Loss of Heterozygosity
MAPK	Mitogen-Activated Protein Kinase
mbp	Megabase Pairs
MCL	Mantle Cell Lymphoma
mCRPC	Metastatic Castration-Resistant Prostate Cancer
MDM2	Murine Double Minute 2
MDMX	Murine Double Minute X
MEK	Mitogen-Activated Protein Kinase Kinase
ML	Machine Learning
MMEJ	Microhomology-Mediated End Joining
miRNA	MicroRNAs
MLH1	MutL Protein Homolog 1
MM	Multiple Myeloma
MMPs	Matrix Metalloproteinases
MRP	Multidrug Resistance-Associated Proteins
MRD	Minimal Residual Disease
mtDNA	Mitochondrial DNA
mTORC2	mTOR Complex 2
NGS	Next-Generation Sequencing
NK	Natural Killer
NMIBC	Non-Muscle-Invasive Bladder Cancer
NSCLC	Non-Small Cell Lung Cancer
PARP1	Poly (ADP-ribose) Polymerase 1
PI3K	Phosphoinositide 3-Kinase
PIP2	Phosphatidylinositol-4,5-bisphosphate
PIP3	Phosphatidylinositol-3,4,5-trisphosphate
PLAT	Tissue Type Plasminogen Activator
PDK1	3-Phosphoinositide-Dependent Protein Kinase 1
POLQ	DNA Polymerase Theta
POLR3K	RNA Polymerase III Subunit K
PP1	Protein Phosphatase
PRC2	Polycomb Repressive Complex 2
PTCL	Peripheral T-Cell Lymphoma
PTEN	Phosphate and Tensin Homolog
PTPRN	Protein Tyrosine Phosphatase Receptor Type N
p21	CDKN1A
pNET	Primitive Neuro-Ectodermal Tumor
RCC	Renal Cell Carcinoma
RIT	Radioimmunotherapy
ROS	Reactive Oxygen Species
RSV	Rous Sarcoma Virus
RTK	Receptor Tyrosine Kinases
sgRNA	Single Guide RNA
SSB	Single-Stranded Breaks
SNP	Single-Nucleotide Polymorphism
SNV	Single Nucleotide Variants
TAM	Tumor-Associated Macrophage
TAA	Tumor-Associated Antigen
TCGA	The Cancer Genome Atlas
TGFβ1	Transforming Growth Factor Beta 1
TNBC	Triple-Negative Breast Cancer
TNF	Tumor Necrosis Factor
TSA	Tumor-Specific Antigen
TS	Targeted Sequencing
TME	Tumor Microenvironment
T-VEC	Talimogene Laherparepvec
VAF	Variant Allele Frequency
VCAN	Versican
VEGF	Vascular Endothelial Growth Factor
WES	Whole Exome Sequencing
WGS	Whole Genome Sequencing
WHO	World Health Organization

## References

[1] Roy P., Saikia B. (2016). Cancer and Cure: A Critical Analysis. Indian J. Cancer.

[2] International Agency for Research on Cancer (IARC) Cancer Factsheets. https://gco.iarc.fr/today/en/fact-sheets-cancers.

[3] Gilbertson R.J. (2011). Mapping Cancer Origins. Cell.

[4] Chatterjee A., Mambo E., Sidransky D. (2006). Mitochondrial DNA Mutations in Human Cancer. Oncogene.

[5] Kim M., Mahmood M., Reznik E., Gammage P.A. (2022). Mitochondrial DNA Is a Major Source of Driver Mutations in Cancer. Trends Cancer.

[6] Porta-Pardo E., Valencia A., Godzik A. (2020). Understanding Oncogenicity of Cancer Driver Genes and Mutations in the Cancer Genomics Era. FEBS Lett..

[7] Lechner J.F., Tesfaigzi J., Gerwin B.I. (1997). Oncogenes and Tumor-Suppressor Genes in Mesothelioma—A Synopsis. Environ. Health Perspect..

[8] Dakal T.C., Dhabhai B., Pant A., Moar K., Chaudhary K., Yadav V., Ranga V., Sharma N.K., Kumar A., Maurya P.K. (2024). Oncogenes and Tumor Suppressor Genes: Functions and Roles in Cancers. MedComm.

[9] Knudson A.G. (1971). Mutation and Cancer: Statistical Study of Retinoblastoma. Proc. Natl. Acad. Sci. USA.

[10] Nichols C.A., Gibson W.J., Brown M.S., Kosmicki J.A., Busanovich J.P., Wei H., Urbanski L.M., Curimjee N., Berger A.C., Gao G.F. (2020). Loss of Heterozygosity of Essential Genes Represents a Widespread Class of Potential Cancer Vulnerabilities. Nat. Commun..

[11] Fluiter K., Housman D., ten Asbroek A.L.M.A., Baas F. (2003). Killing Cancer by Targeting Genes That Cancer Cells Have Lost: Allele-Specific Inhibition, a Novel Approach to the Treatment of Genetic Disorders. Cell. Mol. Life Sci..

[12] Torry D.S., Cooper G.M. (1991). Proto-Oncogenes in Development and Cancer. Am. J. Reprod. Immunol..

[13] Hijiya N., Gewirtz A.M. (1995). Oncogenes, Protooncogenes, and Tumor Suppressor Genes in Acute Myelogenous Leukemia. J. Pediatr. Hematol. Oncol..

[14] Kopelovich L., Shea-Herbert B. (2013). Heritable One-Hit Events Defining Cancer Prevention?. Cell Cycle.

[15] Park S., Supek F., Lehner B. (2021). Higher Order Genetic Interactions Switch Cancer Genes from Two-Hit to One-Hit Drivers. Nat. Commun..

[16] Simatou A., Simatos G., Goulielmaki M., Spandidos D.A., Baliou S., Zoumpourlis V. (2020). Historical Retrospective of the SRC Oncogene and New Perspectives (Review). Mol. Clin. Oncol..

[17] Kontomanolis E.N., Koutras A., Syllaios A., Schizas D., Mastoraki A., Garmpis N., Diakosavvas M., Angelou K., Tsatsaris G., Pagkalos A. (2020). Role of Oncogenes and Tumor-Suppressor Genes in Carcinogenesis: A Review. Anticancer Res..

[18] Roy S., Bondada M.S., Zhang Y., Moffat K., Nair V., Yao Y. (2022). Proviral ALV-LTR Sequence Is Essential for Continued Proliferation of the ALV-Transformed B Cell Line. Int. J. Mol. Sci..

[19] Morsberger L., Pallavajjala A., Long P., Hardy M., Park R., Parish R., Nozari A., Zou Y.S. (2022). HER2 Amplification by Next-Generation Sequencing to Identify HER2-Positive Invasive Breast Cancer with Negative HER2 Immunohistochemistry. Cancer Cell Int..

[20] Zimmerman M.W., Liu Y., He S., Durbin A.D., Abraham B.J., Easton J., Shao Y., Xu B., Zhu S., Zhang X. (2018). MYC Drives a Subset of High-Risk Pediatric Neuroblastomas and Is Activated through Mechanisms Including Enhancer Hijacking and Focal Enhancer Amplification. Cancer Discov..

[21] Gariglio P. (2012). Oncogenes and Tumor Suppressor Genes. Molecular Oncology: Principles and Recent Advances.

[22] Prior I.A., Lewis P.D., Mattos C. (2012). A Comprehensive Survey of Ras Mutations in Cancer. Cancer Res..

[23] Quinlan M.P., Settleman J. (2009). Isoform-Specific Ras Functions in Development and Cancer. Future Oncol..

[24] Hutter S., Bolin S., Weishaupt H., Swartling F. (2017). Modeling and Targeting MYC Genes in Childhood Brain Tumors. Genes.

[25] Ong J.Y., Torres J.Z. (2019). Dissecting the Mechanisms of Cell Division. J. Biol. Chem..

[26] Bischoff J.R., Friedman P.N., Marshak D.R., Prives C., Beach D. (1990). Human P53 Is Phosphorylated by P60-Cdc2 and Cyclin B-Cdc2. Proc. Natl. Acad. Sci. USA.

[27] Canavese M., Santo L., Raje N. (2012). Cyclin Dependent Kinases in Cancer. Cancer Biol. Ther..

[28] Łukasik P., Załuski M., Gutowska I. (2021). Cyclin-Dependent Kinases (CDK) and Their Role in Diseases Development-Review. Int. J. Mol. Sci..

[29] Cao L., Chen F., Yang X., Xu W., Xie J., Yu L. (2014). Phylogenetic Analysis of CDK and Cyclin Proteins in Premetazoan Lineages. BMC Evol. Biol..

[30] Ding L., Cao J., Lin W., Chen H., Xiong X., Ao H., Yu M., Lin J., Cui Q. (2020). The Roles of Cyclin-Dependent Kinases in Cell-Cycle Progression and Therapeutic Strategies in Human Breast Cancer. Int. J. Mol. Sci..

[31] Ghafouri-Fard S., Khoshbakht T., Hussen B.M., Dong P., Gassler N., Taheri M., Baniahmad A., Dilmaghani N.A. (2022). A Review on the Role of Cyclin Dependent Kinases in Cancers. Cancer Cell Int..

[32] Asghar U., Witkiewicz A.K., Turner N.C., Knudsen E.S. (2015). The History and Future of Targeting Cyclin-Dependent Kinases in Cancer Therapy. Nat. Rev. Drug Discov..

[33] Malumbres M., Barbacid M. (2001). To Cycle or Not to Cycle: A Critical Decision in Cancer. Nat. Rev. Cancer.

[34] Topacio B.R., Zatulovskiy E., Cristea S., Xie S., Tambo C.S., Rubin S.M., Sage J., Kõivomägi M., Skotheim J.M. (2019). Cyclin D-Cdk4,6 Drives Cell-Cycle Progression via the Retinoblastoma Protein’s C-Terminal Helix. Mol. Cell.

[35] Pavletich N.P. (1999). Mechanisms of Cyclin-Dependent Kinase Regulation: Structures of Cdks, Their Cyclin Activators, and Cip and INK4 Inhibitors. J. Mol. Biol..

[36] Fagundes R., Teixeira L.K. (2021). Cyclin E/CDK2: DNA Replication, Replication Stress and Genomic Instability. Front. Cell Dev. Biol..

[37] Morgan D.O. (1997). CYCLIN-DEPENDENT KINASES: Engines, Clocks, and Microprocessors. Annu. Rev. Cell Dev. Biol..

[38] Adams P.D. (2001). Regulation of the Retinoblastoma Tumor Suppressor Protein by Cyclin/Cdks. Biochim. Biophys. Acta-Rev. Cancer.

[39] Rubin S.M., Sage J., Skotheim J.M. (2020). Integrating Old and New Paradigms of G1/S Control. Mol. Cell.

[40] Tamrakar S. (2000). Role of PRB Dephosphorylation in Cell Cycle Regulation. Front. Biosci..

[41] Goel S., Bergholz J.S., Zhao J.J. (2022). Targeting CDK4 and CDK6 in Cancer. Nat. Rev. Cancer.

[42] Zhang M., Kim S., Yang H.W. (2023). Non-Canonical Pathway for Rb Inactivation and External Signaling Coordinate Cell-Cycle Entry without CDK4/6 Activity. Nat. Commun..

[43] Hernández Borrero L.J., El-Deiry W.S. (2021). Tumor Suppressor P53: Biology, Signaling Pathways, and Therapeutic Targeting. Biochim. Biophys. Acta-Rev. Cancer.

[44] Wang H., Guo M., Wei H., Chen Y. (2023). Targeting P53 Pathways: Mechanisms, Structures and Advances in Therapy. Signal Transduct. Target. Ther..

[45] Engeland K. (2022). Cell Cycle Regulation: P53-P21-RB Signaling. Cell Death Differ..

[46] Stevaux O., Dyson N.J. (2002). A Revised Picture of the E2F Transcriptional Network and RB Function. Curr. Opin. Cell Biol..

[47] Bieging K.T., Mello S.S., Attardi L.D. (2014). Unravelling Mechanisms of P53-Mediated Tumour Suppression. Nat. Rev. Cancer.

[48] Hou H., Sun D., Zhang X. (2019). The Role of MDM2 Amplification and Overexpression in Therapeutic Resistance of Malignant Tumors. Cancer Cell Int..

[49] Ma M., Hua S., Min X., Wang L., Li J., Wu P., Liang H., Zhang B., Chen X., Xiang S. (2022). P53 Positively Regulates the Proliferation of Hepatic Progenitor Cells Promoted by Laminin-521. Signal Transduct. Target. Ther..

[50] Mihara M., Erster S., Zaika A., Petrenko O., Chittenden T., Pancoska P., Moll U.M. (2003). P53 Has a Direct Apoptogenic Role at the Mitochondria. Mol. Cell.

[51] Vaddavalli P.L., Schumacher B. (2022). The P53 Network: Cellular and Systemic DNA Damage Responses in Cancer and Aging. Trends Genet..

[52] Aubrey B.J., Kelly G.L., Janic A., Herold M.J., Strasser A. (2018). How Does P53 Induce Apoptosis and How Does This Relate to P53-Mediated Tumour Suppression?. Cell Death Differ..

[53] Sinha A., Zou Y., Patel A.S., Yoo S., Jiang F., Sato T., Kong R., Watanabe H., Zhu J., Massion P.P. (2022). Early-Stage Lung Adenocarcinoma MDM2 Genomic Amplification Predicts Clinical Outcome and Response to Targeted Therapy. Cancers.

[54] Martinez-Monleon A., Kryh Öberg H., Gaarder J., Berbegall A.P., Javanmardi N., Djos A., Ussowicz M., Taschner-Mandl S., Ambros I.M., Øra I. (2022). Amplification of CDK4 and MDM2: A Detailed Study of a High-Risk Neuroblastoma Subgroup. Sci. Rep..

[55] Padilla-Nash H.M., McNeil N.E., Yi M., Nguyen Q.-T., Hu Y., Wangsa D., Mack D.L., Hummon A.B., Case C., Cardin E. (2013). Aneuploidy, Oncogene Amplification and Epithelial to Mesenchymal Transition Define Spontaneous Transformation of Murine Epithelial Cells. Carcinogenesis.

[56] Baxter R.C. (2023). Signaling Pathways of the Insulin-like Growth Factor Binding Proteins. Endocr. Rev..

[57] Bianco R., Caputo R., Caputo R., Damiano V., De Placido S., Ficorella C., Agrawal S., Bianco A.R., Ciardiello F., Tortora G. (2004). Combined Targeting of Epidermal Growth Factor Receptor and MDM2 by Gefitinib and Antisense MDM2 Cooperatively Inhibit Hormone-Independent Prostate Cancer. Clin. Cancer Res..

[58] Laroche A., Chaire V., Algeo M.-P., Karanian M., Fourneaux B., Italiano A. (2017). MDM2 Antagonists Synergize with PI3K/MTOR Inhibition in Well-Differentiated/Dedifferentiated Liposarcomas. Oncotarget.

[59] Chibaya L., Karim B., Zhang H., Jones S.N. (2021). Mdm2 Phosphorylation by Akt Regulates the P53 Response to Oxidative Stress to Promote Cell Proliferation and Tumorigenesis. Proc. Natl. Acad. Sci. USA.

[60] Tsvetkov P., Reuven N., Shaul Y. (2010). Ubiquitin-Independent P53 Proteasomal Degradation. Cell Death Differ..

[61] Lossaint G., Horvat A., Gire V., Bačević K., Mrouj K., Charrier-Savournin F., Georget V., Fisher D., Dulić V. (2022). Reciprocal Regulation of P21 and Chk1 Controls the Cyclin D1-RB Pathway to Mediate Senescence Onset after G2 Arrest. J. Cell Sci..

[62] Seong H.-A., Ha H. (2019). Thr55 Phosphorylation of P21 by MPK38/MELK Ameliorates Defects in Glucose, Lipid, and Energy Metabolism in Diet-Induced Obese Mice. Cell Death Dis..

[63] Shibue T., Takeda K., Oda E., Tanaka H., Murasawa H., Takaoka A., Morishita Y., Akira S., Taniguchi T., Tanaka N. (2003). Integral Role of Noxa in P53-Mediated Apoptotic Response. Genes Dev..

[64] Nakano K., Vousden K.H. (2001). PUMA, a Novel Proapoptotic Gene, Is Induced by P53. Mol. Cell.

[65] Wang J., Thomas H.R., Li Z., Yeo N.C., Scott H.E., Dang N., Hossain M.I., Andrabi S.A., Parant J.M. (2021). Puma, Noxa, P53, and P63 Differentially Mediate Stress Pathway Induced Apoptosis. Cell Death Dis..

[66] Basu A. (1998). The Relationship between BcI2, Bax and P53: Consequences for Cell Cycle Progression and Cell Death. Mol. Hum. Reprod..

[67] Xu J., Wang X., Zhu C., Wang K. (2022). A Review of Current Evidence about LncRNA MEG3: A Tumor Suppressor in Multiple Cancers. Front. Cell Dev. Biol..

[68] Guo Y., Pan W., Liu S., Shen Z., Xu Y., Hu L. (2020). ERK/MAPK Signalling Pathway and Tumorigenesis (Review). Exp. Ther. Med..

[69] Nandan M.O., Yang V.W. (2011). An Update on the Biology of RAS/RAF Mutations in Colorectal Cancer. Curr. Color. Cancer Rep..

[70] Campbell S.L., Khosravi-Far R., Rossman K.L., Clark G.J., Der C.J. (1998). Increasing Complexity of Ras Signaling. Oncogene.

[71] Osaka N., Hirota Y., Ito D., Ikeda Y., Kamata R., Fujii Y., Chirasani V.R., Campbell S.L., Takeuchi K., Senda T. (2021). Divergent Mechanisms Activating RAS and Small GTPases Through Post-Translational Modification. Front. Mol. Biosci..

[72] Bahar M.E., Kim H.J., Kim D.R. (2023). Targeting the RAS/RAF/MAPK Pathway for Cancer Therapy: From Mechanism to Clinical Studies. Signal Transduct. Target. Ther..

[73] Molina J.R., Adjei A.A. (2006). The Ras/Raf/MAPK Pathway. J. Thorac. Oncol..

[74] Srdanović S., Wolter M., Trinh C.H., Ottmann C., Warriner S.L., Wilson A.J. (2022). Understanding the Interaction of 14-3-3 Proteins with *h* DMX and *h* DM2: A Structural and Biophysical Study. FEBS J..

[75] Li L., Zhao G.-D., Shi Z., Qi L.-L., Zhou L.-Y., Fu Z.-X. (2016). The Ras/Raf/MEK/ERK Signaling Pathway and Its Role in the Occurrence and Development of HCC. Oncol. Lett..

[76] Boulton T.G., Nye S.H., Robbins D.J., Ip N.Y., Radzlejewska E., Morgenbesser S.D., DePinho R.A., Panayotatos N., Cobb M.H., Yancopoulos G.D. (1991). ERKs: A Family of Protein-Serine/Threonine Kinases That Are Activated and Tyrosine Phosphorylated in Response to Insulin and NGF. Cell.

[77] Huang L., Guo Z., Wang F., Fu L. (2021). KRAS Mutation: From Undruggable to Druggable in Cancer. Signal Transduct. Target. Ther..

[78] Moore A.R., Rosenberg S.C., McCormick F., Malek S. (2020). RAS-Targeted Therapies: Is the Undruggable Drugged?. Nat. Rev. Drug Discov..

[79] Śmiech M., Leszczyński P., Kono H., Wardell C., Taniguchi H. (2020). Emerging BRAF Mutations in Cancer Progression and Their Possible Effects on Transcriptional Networks. Genes.

[80] Cantwell-Dorris E.R., O’Leary J.J., Sheils O.M. (2011). BRAFV600E: Implications for Carcinogenesis and Molecular Therapy. Mol. Cancer Ther..

[81] Śmiech M., Leszczyński P., Wardell C., Poznański P., Pierzchała M., Taniguchi H. (2022). Oncogenic Mutation BRAF V600E Changes Phenotypic Behavior of THLE-2 Liver Cells through Alteration of Gene Expression. Int. J. Mol. Sci..

[82] Jiang X., Wang J., Deng X., Xiong F., Zhang S., Gong Z., Li X., Cao K., Deng H., He Y. (2020). The Role of Microenvironment in Tumor Angiogenesis. J. Exp. Clin. Cancer Res..

[83] Ghalehbandi S., Yuzugulen J., Pranjol M.Z.I., Pourgholami M.H. (2023). The Role of VEGF in Cancer-Induced Angiogenesis and Research Progress of Drugs Targeting VEGF. Eur. J. Pharmacol..

[84] He Y., Sun M.M., Zhang G.G., Yang J., Chen K.S., Xu W.W., Li B. (2021). Targeting PI3K/Akt Signal Transduction for Cancer Therapy. Signal Transduct. Target. Ther..

[85] Vanhaesebroeck B., Guillermet-Guibert J., Graupera M., Bilanges B. (2010). The Emerging Mechanisms of Isoform-Specific PI3K Signalling. Nat. Rev. Mol. Cell Biol..

[86] Liu R., Chen Y., Liu G., Li C., Song Y., Cao Z., Li W., Hu J., Lu C., Liu Y. (2020). PI3K/AKT Pathway as a Key Link Modulates the Multidrug Resistance of Cancers. Cell Death Dis..

[87] Hennessy B.T., Smith D.L., Ram P.T., Lu Y., Mills G.B. (2005). Exploiting the PI3K/AKT Pathway for Cancer Drug Discovery. Nat. Rev. Drug Discov..

[88] Habrowska-Górczyńska D.E., Kozieł M.J., Urbanek K.A., Kowalska K., Piastowska-Ciesielska A.W. (2024). FOXO3a/PI3K/Akt Pathway Participates in the ROS- Induced Apoptosis Triggered by α-ZEL and β-ZEL. Sci. Rep..

[89] Glaviano A., Foo A.S.C., Lam H.Y., Yap K.C.H., Jacot W., Jones R.H., Eng H., Nair M.G., Makvandi P., Geoerger B. (2023). PI3K/AKT/MTOR Signaling Transduction Pathway and Targeted Therapies in Cancer. Mol. Cancer.

[90] Panwar V., Singh A., Bhatt M., Tonk R.K., Azizov S., Raza A.S., Sengupta S., Kumar D., Garg M. (2023). Multifaceted Role of MTOR (Mammalian Target of Rapamycin) Signaling Pathway in Human Health and Disease. Signal Transduct. Target. Ther..

[91] Zou Z., Tao T., Li H., Zhu X. (2020). MTOR Signaling Pathway and MTOR Inhibitors in Cancer: Progress and Challenges. Cell Biosci..

[92] Yang L., Miao L., Liang F., Huang H., Teng X., Li S., Nuriddinov J., Selzer M.E., Hu Y. (2014). The MTORC1 Effectors S6K1 and 4E-BP Play Different Roles in CNS Axon Regeneration. Nat. Commun..

[93] Mukherjee R., Vanaja K.G., Boyer J.A., Gadal S., Solomon H., Chandarlapaty S., Levchenko A., Rosen N. (2021). Regulation of PTEN Translation by PI3K Signaling Maintains Pathway Homeostasis. Mol. Cell.

[94] Zhou J., Du T., Li B., Rong Y., Verkhratsky A., Peng L. (2015). Crosstalk Between MAPK/ERK and PI3K/AKT Signal Pathways During Brain Ischemia/Reperfusion. ASN Neuro.

[95] Dai J., Bercury K.K., Macklin W.B. (2014). Interaction of MTOR and Erk1/2 Signaling to Regulate Oligodendrocyte Differentiation. Glia.

[96] Zhang Y., Wang X. (2020). Targeting the Wnt/β-Catenin Signaling Pathway in Cancer. J. Hematol. Oncol..

[97] Anand A.A., Khan M., Monica V., Kar D. (2023). The Molecular Basis of Wnt/β-Catenin Signaling Pathways in Neurodegenerative Diseases. Int. J. Cell Biol..

[98] Stamos J.L., Weis W.I. (2013). The β-Catenin Destruction Complex. Cold Spring Harb. Perspect. Biol..

[99] MacDonald B.T., He X. (2012). Frizzled and LRP5/6 Receptors for Wnt/β-Catenin Signaling. Cold Spring Harb. Perspect. Biol..

[100] Cadigan K.M., Waterman M.L. (2012). TCF/LEFs and Wnt Signaling in the Nucleus. Cold Spring Harb. Perspect. Biol..

[101] Schlosshauer P.W., Pirog E.C., Levine R.L., Ellenson L.H. (2000). Mutational Analysis of the CTNNB1 and APC Genes in Uterine Endometrioid Carcinoma. Mod. Pathol..

[102] Sehgal P., Lanauze C., Wang X., Hayer K.E., Torres-Diz M., Leu N.A., Sela Y., Stanger B.Z., Lengner C.J., Thomas-Tikhonenko A. (2021). MYC Hyperactivates Wnt Signaling in *APC*/*CTNNB1*-Mutated Colorectal Cancer Cells through MiR-92a–Dependent Repression of *DKK3*. Mol. Cancer Res..

[103] Xue W., Yang L., Chen C., Ashrafizadeh M., Tian Y., Sun R. (2024). Wnt/β-Catenin-Driven EMT Regulation in Human Cancers. Cell. Mol. Life Sci..

[104] Xu L., Zhang L., Hu C., Liang S., Fei X., Yan N., Zhang Y., Zhang F. (2016). WNT Pathway Inhibitor Pyrvinium Pamoate Inhibits the Self-Renewal and Metastasis of Breast Cancer Stem Cells. Int. J. Oncol..

[105] Daisy Precilla S., Biswas I., Kuduvalli S.S., Anitha T.S. (2022). Crosstalk between PI3K/AKT/MTOR and WNT/β-Catenin Signaling in GBM—Could Combination Therapy Checkmate the Collusion?. Cell. Signal..

[106] Fleming-de-Moraes C.D., Rocha M.R., Tessmann J.W., de Araujo W.M., Morgado-Diaz J.A. (2022). Crosstalk between PI3K/Akt and Wnt/β-Catenin Pathways Promote Colorectal Cancer Progression Regardless of Mutational Status. Cancer Biol. Ther..

[107] Langhammer T.-S., Roolf C., Krohn S., Kretzschmar C., Huebner R., Rolfs A., Freund M., Junghanss C. (2013). PI3K/Akt Signaling Interacts with Wnt/β-Catenin Signaling But Does Not Induce an Accumulation of β-Catenin in the Nucleus of Acute Lymphoblastic Leukemia Cell Lines. Blood.

[108] Koujah L., Madavaraju K., Agelidis A.M., Patil C.D., Shukla D. (2021). Heparanase-Induced Activation of AKT Stabilizes β-Catenin and Modulates Wnt/β-Catenin Signaling during Herpes Simplex Virus 1 Infection. MBio.

[109] Fang D., Hawke D., Zheng Y., Xia Y., Meisenhelder J., Nika H., Mills G.B., Kobayashi R., Hunter T., Lu Z. (2007). Phosphorylation of β-Catenin by AKT Promotes β-Catenin Transcriptional Activity. J. Biol. Chem..

[110] Jeong W.-J., Ro E.J., Choi K.-Y. (2018). Interaction between Wnt/β-Catenin and RAS-ERK Pathways and an Anti-Cancer Strategy via Degradations of β-Catenin and RAS by Targeting the Wnt/β-Catenin Pathway. npj Precis. Oncol..

[111] Zhang Y., Pizzute T., Pei M. (2014). A Review of Crosstalk between MAPK and Wnt Signals and Its Impact on Cartilage Regeneration. Cell Tissue Res..

[112] Sharma S., Kelly T.K., Jones P.A. (2010). Epigenetics in Cancer. Carcinogenesis.

[113] Jones P.A., Baylin S.B. (2002). The Fundamental Role of Epigenetic Events in Cancer. Nat. Rev. Genet..

[114] Egger G., Liang G., Aparicio A., Jones P.A. (2004). Epigenetics in Human Disease and Prospects for Epigenetic Therapy. Nature.

[115] Sonar S., Nyahatkar S., Kalele K., Adhikari M.D. (2024). Role of DNA Methylation in Cancer Development and Its Clinical Applications. Clin. Transl. Discov..

[116] Lakshminarasimhan R., Liang G. (2016). The Role of DNA Methylation in Cancer. DNA Methyltransferases-Role and Function.

[117] Stojchevski R., Velichkovikj S., Arsov T. (2023). Genetic and Epigenetic Basis of Obesity-Induced Inflammation and Diabetes. Obesity, Diabetes and Inflammation: Molecular Mechanisms and Clinical Management.

[118] Nishiyama A., Nakanishi M. (2021). Navigating the DNA Methylation Landscape of Cancer. Trends Genet..

[119] Pan Y., Liu G., Zhou F., Su B., Li Y. (2018). DNA Methylation Profiles in Cancer Diagnosis and Therapeutics. Clin. Exp. Med..

[120] Ehrlich M. (2019). DNA Hypermethylation in Disease: Mechanisms and Clinical Relevance. Epigenetics.

[121] Niv Y. (2007). Microsatellite Instability and MLH1 Promoter Hypermethylation in Colorectal Cancer. World J. Gastroenterol..

[122] Oubaddou Y., Oukabli M., Fenniche S., Elktaibi A., Elochi M.R., Al Bouzidi A., Qmichou Z., Dakka N., Diorio C., Richter A. (2023). BRCA1 Promoter Hypermethylation in Malignant Breast Tumors and in the Histologically Normal Adjacent Tissues to the Tumors: Exploring Its Potential as a Biomarker and Its Clinical Significance in a Translational Approach. Genes.

[123] Tramontano A., Boffo F.L., Russo G., De Rosa M., Iodice I., Pezone A. (2020). Methylation of the Suppressor Gene P16INK4a: Mechanism and Consequences. Biomolecules.

[124] Besselink N., Keijer J., Vermeulen C., Boymans S., de Ridder J., van Hoeck A., Cuppen E., Kuijk E. (2023). The Genome-Wide Mutational Consequences of DNA Hypomethylation. Sci. Rep..

[125] Choi J.-Y., James S.R., Link P.A., McCann S.E., Hong C.-C., Davis W., Nesline M.K., Ambrosone C.B., Karpf A.R. (2009). Association between Global DNA Hypomethylation in Leukocytes and Risk of Breast Cancer. Carcinogenesis.

[126] Tripathi K., Goel A., Singhai A., Garg M. (2021). Promoter Hypomethylation as Potential Confounder of Ras Gene Overexpression and Their Clinical Significance in Subsets of Urothelial Carcinoma of Bladder. Mol. Biol. Rep..

[127] Ehrlich M. (2009). Dna Hypomethylation In Cancer Cells. Epigenomics.

[128] Fain J.S., Loriot A., Diacofotaki A., Van Tongelen A., De Smet C. (2021). Transcriptional Overlap Links DNA Hypomethylation with DNA Hypermethylation at Adjacent Promoters in Cancer. Sci. Rep..

[129] Zhao Z., Shilatifard A. (2019). Epigenetic Modifications of Histones in Cancer. Genome Biol..

[130] Miller J.L., Grant P.A. (2013). The Role of DNA Methylation and Histone Modifications in Transcriptional Regulation in Humans. Subcell. Biochem..

[131] Kornberg R.D. (1974). Chromatin Structure: A Repeating Unit of Histones and DNA. Science.

[132] Alaskhar Alhamwe B., Khalaila R., Wolf J., von Bülow V., Harb H., Alhamdan F., Hii C.S., Prescott S.L., Ferrante A., Renz H. (2018). Histone Modifications and Their Role in Epigenetics of Atopy and Allergic Diseases. Allergy Asthma Clin. Immunol..

[133] Arif K.M.T., Elliott E.K., Haupt L.M., Griffiths L.R. (2020). Regulatory Mechanisms of Epigenetic MiRNA Relationships in Human Cancer and Potential as Therapeutic Targets. Cancers.

[134] Reynolds P.A., Sigaroudinia M., Zardo G., Wilson M.B., Benton G.M., Miller C.J., Hong C., Fridlyand J., Costello J.F., Tlsty T.D. (2006). Tumor Suppressor P16INK4A Regulates Polycomb-Mediated DNA Hypermethylation in Human Mammary Epithelial Cells. J. Biol. Chem..

[135] Laugesen A., Højfeldt J.W., Helin K. (2016). Role of the Polycomb Repressive Complex 2 (PRC2) in Transcriptional Regulation and Cancer. Cold Spring Harb. Perspect. Med..

[136] Albero R., Enjuanes A., Demajo S., Castellano G., Pinyol M., García N., Capdevila C., Clot G., Suárez-Cisneros H., Shimada M. (2018). Cyclin D1 Overexpression Induces Global Transcriptional Downregulation in Lymphoid Neoplasms. J. Clin. Investig..

[137] Braile M., Luciano N., Carlomagno D., Salvatore G., Orlandella F.M. (2024). Insight into the Role of the MiR-584 Family in Human Cancers. Int. J. Mol. Sci..

[138] Calin G.A., Dumitru C.D., Shimizu M., Bichi R., Zupo S., Noch E., Aldler H., Rattan S., Keating M., Rai K. (2002). Frequent Deletions and Down-Regulation of Micro- RNA Genes *MiR15* and *MiR16* at 13q14 in Chronic Lymphocytic Leukemia. Proc. Natl. Acad. Sci. USA.

[139] Torres A., Torres K., Pesci A., Ceccaroni M., Paszkowski T., Cassandrini P., Zamboni G., Maciejewski R. (2012). Deregulation of MiR-100, MiR-99a and MiR-199b in Tissues and Plasma Coexists with Increased Expression of MTOR Kinase in Endometrioid Endometrial Carcinoma. BMC Cancer.

[140] Fan D., Ren B., Yang X., Liu J., Zhang Z. (2016). Upregulation of MiR-501-5p Activates the Wnt/β-Catenin Signaling Pathway and Enhances Stem Cell-like Phenotype in Gastric Cancer. J. Exp. Clin. Cancer Res..

[141] Zhang C., Kang C., Wang P., Cao Y., Lv Z., Yu S., Wang G., Zhang A., Jia Z., Han L. (2011). MicroRNA-221 and -222 Regulate Radiation Sensitivity by Targeting the PTEN Pathway. Int. J. Radiat. Oncol..

[142] Ma Y., Shen N., Wicha M.S., Luo M. (2021). The Roles of the Let-7 Family of MicroRNAs in the Regulation of Cancer Stemness. Cells.

[143] Zhu L., Jiang M., Wang H., Sun H., Zhu J., Zhao W., Fang Q., Yu J., Chen P., Wu S. (2021). A Narrative Review of Tumor Heterogeneity and Challenges to Tumor Drug Therapy. Ann. Transl. Med..

[144] Dagogo-Jack I., Shaw A.T. (2018). Tumour Heterogeneity and Resistance to Cancer Therapies. Nat. Rev. Clin. Oncol..

[145] Liu J., Dang H., Wang X.W. (2018). The Significance of Intertumor and Intratumor Heterogeneity in Liver Cancer. Exp. Mol. Med..

[146] Jamal-Hanjani M., Wilson G.A., McGranahan N., Birkbak N.J., Watkins T.B.K., Veeriah S., Shafi S., Johnson D.H., Mitter R., Rosenthal R. (2017). Tracking the Evolution of Non–Small-Cell Lung Cancer. N. Engl. J. Med..

[147] Campbell P.J., Yachida S., Mudie L.J., Stephens P.J., Pleasance E.D., Stebbings L.A., Morsberger L.A., Latimer C., McLaren S., Lin M.-L. (2010). The Patterns and Dynamics of Genomic Instability in Metastatic Pancreatic Cancer. Nature.

[148] Shi Z.-D., Sun Z., Zhu Z.-B., Liu X., Chen J.-Z., Hao L., Zhu J.-F., Pang K., Wu D., Dong Y. (2023). Integrated Single-Cell and Spatial Transcriptomic Profiling Reveals Higher Intratumour Heterogeneity and Epithelial-Fibroblast Interactions in Recurrent Bladder Cancer. Clin. Transl. Med..

[149] Morrissy A.S., Cavalli F.M.G., Remke M., Ramaswamy V., Shih D.J.H., Holgado B.L., Farooq H., Donovan L.K., Garzia L., Agnihotri S. (2017). Spatial Heterogeneity in Medulloblastoma. Nat. Genet..

[150] Andor N., Graham T.A., Jansen M., Xia L.C., Aktipis C.A., Petritsch C., Ji H.P., Maley C.C. (2016). Pan-Cancer Analysis of the Extent and Consequences of Intratumor Heterogeneity. Nat. Med..

[151] McGranahan N., Swanton C. (2017). Clonal Heterogeneity and Tumor Evolution: Past, Present, and the Future. Cell.

[152] Fu F., Nowak M.A., Bonhoeffer S. (2015). Spatial Heterogeneity in Drug Concentrations Can Facilitate the Emergence of Resistance to Cancer Therapy. PLoS Comput. Biol..

[153] Zhou S., Zheng J., Zhai W., Chen Y. (2023). Spatio-Temporal Heterogeneity in Cancer Evolution and Tumor Microenvironment of Renal Cell Carcinoma with Tumor Thrombus. Cancer Lett..

[154] de Oliveira D., Dall’Oglio M.F., Reis S.T., Zerati M., Souza I.C., Leite K.R., Srougi M. (2014). Chromosome 9p Deletions Are an Independent Predictor of Tumor Progression Following Nephrectomy in Patients with Localized Clear Cell Renal Cell Carcinoma. Urol. Oncol. Semin. Orig. Investig..

[155] Narimatsu T., Matsuura K., Nakada C., Tsukamoto Y., Hijiya N., Kai T., Inoue T., Uchida T., Nomura T., Sato F. (2015). Downregulation of NDUFB6 Due to 9p24.1-p13.3 Loss Is Implicated in Metastatic Clear Cell Renal Cell Carcinoma. Cancer Med..

[156] Whitlock B.D., Leslie E.M. (2020). Efflux Transporters in Anti-Cancer Drug Resistance: Molecular and Functional Identification and Characterization of Multidrug Resistance Proteins (MRPs/ABCCs). Drug Efflux Pumps in Cancer Resistance Pathways: From Molecular Recognition and Characterization to Possible Inhibition Strategies in Chemotherapy.

[157] Mansoori B., Mohammadi A., Davudian S., Shirjang S., Baradaran B. (2017). The Different Mechanisms of Cancer Drug Resistance: A Brief Review. Adv. Pharm. Bull..

[158] Ashique S., Bhowmick M., Pal R., Khatoon H., Kumar P., Sharma H., Garg A., Kumar S., Das U. (2024). Multi Drug Resistance in Colorectal Cancer-Approaches to Overcome, Advancements and Future Success. Adv. Cancer Biol.-Metastasis.

[159] Wallbillich N.J., Lu H. (2023). Role of C-Myc in Lung Cancer: Progress, Challenges, and Prospects. Chin. Med. J. Pulm. Crit. Care Med..

[160] Al-Abdulla R., Perez-Silva L., Lozano E., Macias R.I.R., Herraez E., Abad M., Segues N., Bujanda L., Briz O., Marin J.J.G. (2020). Sensitizing Gastric Adenocarcinoma to Chemotherapy by Pharmacological Manipulation of Drug Transporters. Biochem. Pharmacol..

[161] Mao Q., Unadkat J.D. (2015). Role of the Breast Cancer Resistance Protein (BCRP/ABCG2) in Drug Transport—An Update. AAPS J..

[162] Uceda-Castro R., Margarido A.S., Song J.-Y., de Gooijer M.C., Messal H.A., Chambers C.R., Nobis M., Çitirikkaya C.H., Hahn K., Seinstra D. (2023). BCRP Drives Intrinsic Chemoresistance in Chemotherapy-Naïve Breast Cancer Brain Metastasis. Sci. Adv..

[163] Belan O., Sebald M., Adamowicz M., Anand R., Vancevska A., Neves J., Grinkevich V., Hewitt G., Segura-Bayona S., Bellelli R. (2022). POLQ Seals Post-Replicative SsDNA Gaps to Maintain Genome Stability in BRCA-Deficient Cancer Cells. Mol. Cell.

[164] Lemée F., Bergoglio V., Fernandez-Vidal A., Machado-Silva A., Pillaire M.-J., Bieth A., Gentil C., Baker L., Martin A.-L., Leduc C. (2010). *DNA Polymerase* θ up-Regulation Is Associated with Poor Survival in Breast Cancer, Perturbs DNA Replication, and Promotes Genetic Instability. Proc. Natl. Acad. Sci. USA.

[165] Higgins G.S., Harris A.L., Prevo R., Helleday T., McKenna W.G., Buffa F.M. (2010). Overexpression of POLQ Confers a Poor Prognosis in Early Breast Cancer Patients. Oncotarget.

[166] Ray Chaudhuri A., Nussenzweig A. (2017). The Multifaceted Roles of PARP1 in DNA Repair and Chromatin Remodelling. Nat. Rev. Mol. Cell Biol..

[167] Rojo F., García-Parra J., Zazo S., Tusquets I., Ferrer-Lozano J., Menendez S., Eroles P., Chamizo C., Servitja S., Ramírez-Merino N. (2012). Nuclear PARP-1 Protein Overexpression Is Associated with Poor Overall Survival in Early Breast Cancer. Ann. Oncol..

[168] Gilabert M., Launay S., Ginestier C., Bertucci F., Audebert S., Pophillat M., Toiron Y., Baudelet E., Finetti P., Noguchi T. (2014). Poly(ADP-Ribose) Polymerase 1 (PARP1) Overexpression in Human Breast Cancer Stem Cells and Resistance to Olaparib. PLoS ONE.

[169] Ossovskaya V., Koo I.C., Kaldjian E.P., Alvares C., Sherman B.M. (2010). Upregulation of Poly (ADP-Ribose) Polymerase-1 (PARP1) in Triple-Negative Breast Cancer and Other Primary Human Tumor Types. Genes Cancer.

[170] Mazzotta A., Partipilo G., De Summa S., Giotta F., Simone G., Mangia A. (2016). Nuclear PARP1 Expression and Its Prognostic Significance in Breast Cancer Patients. Tumor Biol..

[171] Wang F., Gouttia O.G., Wang L., Peng A. (2022). PARP1 Upregulation in Recurrent Oral Cancer and Treatment Resistance. Front. Cell Dev. Biol..

[172] Zatreanu D., Robinson H.M.R., Alkhatib O., Boursier M., Finch H., Geo L., Grande D., Grinkevich V., Heald R.A., Langdon S. (2021). Polθ Inhibitors Elicit BRCA-Gene Synthetic Lethality and Target PARP Inhibitor Resistance. Nat. Commun..

[173] Oh G., Wang A., Wang L., Li J., Werba G., Weissinger D., Zhao E., Dhara S., Hernandez R.E., Ackermann A. (2023). POLQ Inhibition Elicits an Immune Response in Homologous Recombination–Deficient Pancreatic Adenocarcinoma via CGAS/STING Signaling. J. Clin. Investig..

[174] Liu P., Hao L., Liu M., Hu S. (2023). Glutathione-Responsive and -Exhausting Metal Nanomedicines for Robust Synergistic Cancer Therapy. Front. Bioeng. Biotechnol..

[175] Niu B., Liao K., Zhou Y., Wen T., Quan G., Pan X., Wu C. (2021). Application of Glutathione Depletion in Cancer Therapy: Enhanced ROS-Based Therapy, Ferroptosis, and Chemotherapy. Biomaterials.

[176] Forman H.J., Zhang H., Rinna A. (2009). Glutathione: Overview of Its Protective Roles, Measurement, and Biosynthesis. Mol. Asp. Med..

[177] Hu J., Liu S. (2020). Modulating Intracellular Oxidative Stress via Engineered Nanotherapeutics. J. Control. Release.

[178] Traverso N., Ricciarelli R., Nitti M., Marengo B., Furfaro A.L., Pronzato M.A., Marinari U.M., Domenicotti C. (2013). Role of Glutathione in Cancer Progression and Chemoresistance. Oxid. Med. Cell. Longev..

[179] Kerr E.M., Gaude E., Turrell F.K., Frezza C., Martins C.P. (2016). Mutant Kras Copy Number Defines Metabolic Reprogramming and Therapeutic Susceptibilities. Nature.

[180] Su Y., Zhao B., Zhou L., Zhang Z., Shen Y., Lv H., AlQudsy L.H.H., Shang P. (2020). Ferroptosis, a Novel Pharmacological Mechanism of Anti-Cancer Drugs. Cancer Lett..

[181] Yan H., Talty R., Jain A., Cai Y., Zheng J., Shen X., Muca E., Paty P.B., Bosenberg M.W., Khan S.A. (2023). Discovery of Decreased Ferroptosis in Male Colorectal Cancer Patients with KRAS Mutations. Redox Biol..

[182] Pupo E., Avanzato D., Middonti E., Bussolino F., Lanzetti L. (2019). KRAS-Driven Metabolic Rewiring Reveals Novel Actionable Targets in Cancer. Front. Oncol..

[183] Datta N., Chakraborty S., Basu M., Ghosh M.K. (2020). Tumor Suppressors Having Oncogenic Functions: The Double Agents. Cells.

[184] Stein Y., Rotter V., Aloni-Grinstein R. (2019). Gain-of-Function Mutant P53: All the Roads Lead to Tumorigenesis. Int. J. Mol. Sci..

[185] Kadosh E., Snir-Alkalay I., Venkatachalam A., May S., Lasry A., Elyada E., Zinger A., Shaham M., Vaalani G., Mernberger M. (2020). The Gut Microbiome Switches Mutant P53 from Tumour-Suppressive to Oncogenic. Nature.

[186] McMahon S.B. (2014). MYC and the Control of Apoptosis. Cold Spring Harb. Perspect. Med..

[187] Principe D.R., Doll J.A., Bauer J., Jung B., Munshi H.G., Bartholin L., Pasche B., Lee C., Grippo P.J. (2014). TGF-β: Duality of Function Between Tumor Prevention and Carcinogenesis. JNCI J. Natl. Cancer Inst..

[188] Satam H., Joshi K., Mangrolia U., Waghoo S., Zaidi G., Rawool S., Thakare R.P., Banday S., Mishra A.K., Das G. (2023). Next-Generation Sequencing Technology: Current Trends and Advancements. Biology.

[189] Qin D. (2019). Next-Generation Sequencing and Its Clinical Application. Cancer Biol. Med..

[190] Sanger F., Nicklen S., Coulson A.R. (1977). DNA Sequencing with Chain-Terminating Inhibitors. Proc. Natl. Acad. Sci. USA.

[191] Hu T., Chitnis N., Monos D., Dinh A. (2021). Next-Generation Sequencing Technologies: An Overview. Hum. Immunol..

[192] Wang Z., Gerstein M., Snyder M. (2009). RNA-Seq: A Revolutionary Tool for Transcriptomics. Nat. Rev. Genet..

[193] Lightbody G., Haberland V., Browne F., Taggart L., Zheng H., Parkes E., Blayney J.K. (2019). Review of Applications of High-Throughput Sequencing in Personalized Medicine: Barriers and Facilitators of Future Progress in Research and Clinical Application. Brief. Bioinform..

[194] Shendure J., Balasubramanian S., Church G.M., Gilbert W., Rogers J., Schloss J.A., Waterston R.H. (2017). DNA Sequencing at 40: Past, Present and Future. Nature.

[195] Beedanagari S., John K. (2014). Next Generation Sequencing. Encyclopedia of Toxicology.

[196] Logsdon G.A., Vollger M.R., Eichler E.E. (2020). Long-Read Human Genome Sequencing and Its Applications. Nat. Rev. Genet..

[197] Bentley D.R., Balasubramanian S., Swerdlow H.P., Smith G.P., Milton J., Brown C.G., Hall K.P., Evers D.J., Barnes C.L., Bignell H.R. (2008). Accurate Whole Human Genome Sequencing Using Reversible Terminator Chemistry. Nature.

[198] Choo Z.-N., Behr J.M., Deshpande A., Hadi K., Yao X., Tian H., Takai K., Zakusilo G., Rosiene J., Da Cruz Paula A. (2023). Most Large Structural Variants in Cancer Genomes Can Be Detected without Long Reads. Nat. Genet..

[199] Bagger F.O., Borgwardt L., Jespersen A.S., Hansen A.R., Bertelsen B., Kodama M., Nielsen F.C. (2024). Whole Genome Sequencing in Clinical Practice. BMC Med. Genom..

[200] Souche E., Beltran S., Brosens E., Belmont J.W., Fossum M., Riess O., Gilissen C., Ardeshirdavani A., Houge G., van Gijn M. (2022). Recommendations for Whole Genome Sequencing in Diagnostics for Rare Diseases. Eur. J. Hum. Genet..

[201] The Lancet Oncology (2024). Incorporating Whole-Genome Sequencing into Cancer Care. Lancet Oncol..

[202] Kinnersley B., Sud A., Everall A., Cornish A.J., Chubb D., Culliford R., Gruber A.J., Lärkeryd A., Mitsopoulos C., Wedge D. (2024). Analysis of 10,478 Cancer Genomes Identifies Candidate Driver Genes and Opportunities for Precision Oncology. Nat. Genet..

[203] Iglesias A., Anyane-Yeboa K., Wynn J., Wilson A., Truitt Cho M., Guzman E., Sisson R., Egan C., Chung W.K. (2014). The Usefulness of Whole-Exome Sequencing in Routine Clinical Practice. Genet. Med..

[204] Rabbani B., Tekin M., Mahdieh N. (2014). The Promise of Whole-Exome Sequencing in Medical Genetics. J. Hum. Genet..

[205] Modai S., Shomron N. (2016). Molecular Risk Factors for Schizophrenia. Trends Mol. Med..

[206] Ganatra H., Tan J.K., Simmons A., Bigogno C.M., Khurana V., Ghose A., Ghosh A., Mahajan I., Boussios S., Maniam A. (2024). Applying Whole-Genome and Whole-Exome Sequencing in Breast Cancer: A Review of the Landscape. Breast Cancer.

[207] Hamdi Y., Boujemaa M., Ben Rekaya M., Ben Hamda C., Mighri N., El Benna H., Mejri N., Labidi S., Daoud N., Naouali C. (2018). Family Specific Genetic Predisposition to Breast Cancer: Results from Tunisian Whole Exome Sequenced Breast Cancer Cases. J. Transl. Med..

[208] Pei X.M., Yeung M.H.Y., Wong A.N.N., Tsang H.F., Yu A.C.S., Yim A.K.Y., Wong S.C.C. (2023). Targeted Sequencing Approach and Its Clinical Applications for the Molecular Diagnosis of Human Diseases. Cells.

[209] Bewicke-Copley F., Arjun Kumar E., Palladino G., Korfi K., Wang J. (2019). Applications and Analysis of Targeted Genomic Sequencing in Cancer Studies. Comput. Struct. Biotechnol. J..

[210] Tawana K., Wang J., Renneville A., Bödör C., Hills R., Loveday C., Savic A., Van Delft F.W., Treleaven J., Georgiades P. (2015). Disease Evolution and Outcomes in Familial AML with Germline CEBPA Mutations. Blood.

[211] Shin H.-T., Choi Y.-L., Yun J.W., Kim N.K.D., Kim S.-Y., Jeon H.J., Nam J.-Y., Lee C., Ryu D., Kim S.C. (2017). Prevalence and Detection of Low-Allele-Fraction Variants in Clinical Cancer Samples. Nat. Commun..

[212] Yates L.R., Gerstung M., Knappskog S., Desmedt C., Gundem G., Van Loo P., Aas T., Alexandrov L.B., Larsimont D., Davies H. (2015). Subclonal Diversification of Primary Breast Cancer Revealed by Multiregion Sequencing. Nat. Med..

[213] Padma V.V. (2015). An Overview of Targeted Cancer Therapy. BioMedicine.

[214] Shuel S.L. (2022). Targeted Cancer Therapies: Clinical Pearls for Primary Care. Can. Fam. Physician.

[215] Min H.-Y., Lee H.-Y. (2022). Molecular Targeted Therapy for Anticancer Treatment. Exp. Mol. Med..

[216] PETERS G.J. (2019). From ‘Targeted Therapy’ to Targeted Therapy. Anticancer Res..

[217] Charlton P., Spicer J. (2016). Targeted Therapy in Cancer. Medicine.

[218] Lee Y.T., Tan Y.J., Oon C.E. (2018). Molecular Targeted Therapy: Treating Cancer with Specificity. Eur. J. Pharmacol..

[219] Liu G.-H., Chen T., Zhang X., Ma X.-L., Shi H.-S. (2022). Small Molecule Inhibitors Targeting the Cancers. MedComm.

[220] Hertzman Johansson C., Egyhazi Brage S. (2014). BRAF Inhibitors in Cancer Therapy. Pharmacol. Ther..

[221] Straussman R., Morikawa T., Shee K., Barzily-Rokni M., Qian Z.R., Du J., Davis A., Mongare M.M., Gould J., Frederick D.T. (2012). Tumour Micro-Environment Elicits Innate Resistance to RAF Inhibitors through HGF Secretion. Nature.

[222] Asić K. (2016). Dominant Mechanisms of Primary Resistance Differ from Dominant Mechanisms of Secondary Resistance to Targeted Therapies. Crit. Rev. Oncol. Hematol..

[223] Balik K., Modrakowska P., Maj M., Kaźmierski Ł., Bajek A. (2019). Limitations of Molecularly Targeted Therapy. Med. Res. J..

[224] Zahavi D., Weiner L. (2020). Monoclonal Antibodies in Cancer Therapy. Antibodies.

[225] Singh S., Kumar N.K., Dwiwedi P., Charan J., Kaur R., Sidhu P., Chugh V.K. (2018). Monoclonal Antibodies: A Review. Curr. Clin. Pharmacol..

[226] Kumar M., Jalota A., Sahu S.K., Haque S. (2024). Therapeutic Antibodies for the Prevention and Treatment of Cancer. J. Biomed. Sci..

[227] Pincetic A., Bournazos S., DiLillo D.J., Maamary J., Wang T.T., Dahan R., Fiebiger B.-M., Ravetch J.V. (2014). Type I and Type II Fc Receptors Regulate Innate and Adaptive Immunity. Nat. Immunol..

[228] Bournazos S., Wang T.T., Dahan R., Maamary J., Ravetch J.V. (2017). Signaling by Antibodies: Recent Progress. Annu. Rev. Immunol..

[229] Early Breast Cancer Trialists’ Collaborative Group (EBCTCG) (2021). Trastuzumab for Early-Stage, HER2-Positive Breast Cancer: A Meta-Analysis of 13,864 Women in Seven Randomised Trials. Lancet Oncol..

[230] Swain S.M., Shastry M., Hamilton E. (2023). Targeting HER2-Positive Breast Cancer: Advances and Future Directions. Nat. Rev. Drug Discov..

[231] Zhou J., Ji Q., Li Q. (2021). Resistance to Anti-EGFR Therapies in Metastatic Colorectal Cancer: Underlying Mechanisms and Reversal Strategies. J. Exp. Clin. Cancer Res..

[232] Kasi P.M., Afable M.G., Herting C., Lukanowski M., Jin Z. (2023). Anti-EGFR Antibodies in the Management of Advanced Colorectal Cancer. Oncologist.

[233] Gordeev A., Vaal A., Puchkova M., Smirnova I., Doronin A., Znobishcheva A., Zhmudanova D., Aleksandrov A., Sukchev M., Imyanitov E. (2024). Preclinical Comparison of Prolgolimab, Pembrolizumab and Nivolumab. Sci. Rep..

[234] McMahon D.J., McLaughlin R., Naidoo J. (2024). Is Immunotherapy Beneficial in Patients with Oncogene-Addicted Non-Small Cell Lung Cancers? A Narrative Review. Cancers.

[235] Rotte A. (2019). Combination of CTLA-4 and PD-1 Blockers for Treatment of Cancer. J. Exp. Clin. Cancer Res..

[236] Shahid K., Khalife M., Dabney R., Phan A.T. (2019). Immunotherapy and Targeted Therapy—The New Roadmap in Cancer Treatment. Ann. Transl. Med..

[237] McLean L., Leal J.L., Solomon B.J., John T. (2021). Immunotherapy in Oncogene Addicted Non-Small Cell Lung Cancer. Transl. Lung Cancer Res..

[238] Zhang J., Vokes N., Li M., Xu J., Bai H., Wang J., Wang Z., Zhang J. (2024). Overcoming EGFR-TKI Resistance by Targeting the Tumor Microenvironment. Chin. Med. J. Pulm. Crit. Care Med..

[239] Dailah H.G., Hommdi A.A., Koriri M.D., Algathlan E.M., Mohan S. (2024). Potential Role of Immunotherapy and Targeted Therapy in the Treatment of Cancer: A Contemporary Nursing Practice. Heliyon.

[240] Otano I., Ucero A.C., Zugazagoitia J., Paz-Ares L. (2023). At the Crossroads of Immunotherapy for Oncogene-Addicted Subsets of NSCLC. Nat. Rev. Clin. Oncol..

[241] Garcia J., Hurwitz H.I., Sandler A.B., Miles D., Coleman R.L., Deurloo R., Chinot O.L. (2020). Bevacizumab (Avastin^®^) in Cancer Treatment: A Review of 15 Years of Clinical Experience and Future Outlook. Cancer Treat. Rev..

[242] Bodet-Milin C., Kraeber-Bodéré F., Eugène T., Guérard F., Gaschet J., Bailly C., Mougin M., Bourgeois M., Faivre-Chauvet A., Chérel M. (2016). Radioimmunotherapy for Treatment of Acute Leukemia. Semin. Nucl. Med..

[243] Larson S.M., Carrasquillo J.A., Cheung N.-K.V., Press O.W. (2015). Radioimmunotherapy of Human Tumours. Nat. Rev. Cancer.

[244] Abbasi A., Dadashpour M., Alipourfard I. (2021). Calculation of Radium-223 and Actinium-225 α-Emitter Radiopharmaceuticals Dose Rates in Treatment of Metastatic Castration-Resistant Prostate Cancer. J. Cancer Res. Ther..

[245] Cremonesi M., Ferrari M., Grana C.M., Vanazzi A., Stabin M., Bartolomei M., Papi S., Prisco G., Martinelli G., Paganelli G. (2007). High-Dose Radioimmunotherapy with 90Y-Ibritumomab Tiuxetan: Comparative Dosimetric Study for Tailored Treatment. J. Nucl. Med..

[246] Guo Y., Parry J.J., Laforest R., Rogers B.E., Anderson C.J. (2013). The Role of P53 in Combination Radioimmunotherapy with ^64^Cu-DOTA-Cetuximab and Cisplatin in a Mouse Model of Colorectal Cancer. J. Nucl. Med..

[247] Fu Z., Li S., Han S., Shi C., Zhang Y. (2022). Antibody Drug Conjugate: The “Biological Missile” for Targeted Cancer Therapy. Signal Transduct. Target. Ther..

[248] Hurwitz J., Haggstrom L.R., Lim E. (2023). Antibody–Drug Conjugates: Ushering in a New Era of Cancer Therapy. Pharmaceutics.

[249] Baron J., Wang E.S. (2018). Gemtuzumab Ozogamicin for the Treatment of Acute Myeloid Leukemia. Expert Rev. Clin. Pharmacol..

[250] Gogia P., Ashraf H., Bhasin S., Xu Y. (2023). Antibody–Drug Conjugates: A Review of Approved Drugs and Their Clinical Level of Evidence. Cancers.

[251] Levine B.L., Miskin J., Wonnacott K., Keir C. (2017). Global Manufacturing of CAR T Cell Therapy. Mol. Ther.-Methods Clin. Dev..

[252] De Marco R.C., Monzo H.J., Ojala P.M. (2023). CAR T Cell Therapy: A Versatile Living Drug. Int. J. Mol. Sci..

[253] Majzner R.G., Mackall C.L. (2019). Clinical Lessons Learned from the First Leg of the CAR T Cell Journey. Nat. Med..

[254] Goyco Vera D., Waghela H., Nuh M., Pan J., Lulla P. (2024). Approved CAR-T Therapies Have Reproducible Efficacy and Safety in Clinical Practice. Hum. Vaccines Immunother..

[255] Keefe D.M.K., Bateman E.H. (2019). Potential Successes and Challenges of Targeted Cancer Therapies. JNCI Monogr..

[256] Khan S.U., Fatima K., Aisha S., Malik F. (2024). Unveiling the Mechanisms and Challenges of Cancer Drug Resistance. Cell Commun. Signal..

[257] American Cancer Society Managing Cancer-Related Side Effects. https://www.cancer.org/cancer/managing-cancer/side-effects.html.

[258] Kiss B., Borbély J. (2023). Business Risk Mitigation in the Development Process of New Monoclonal Antibody Drug Conjugates for Cancer Treatment. Pharmaceutics.

[259] Dropulić B. (2024). CAR-T and Cellular Gene Therapies Are Too Expensive. Nat. Med..

[260] Singh D., Dhiman V.K., Pandey M., Dhiman V.K., Sharma A., Pandey H., Verma S.K., Pandey R. (2024). Personalized Medicine: An Alternative for Cancer Treatment. Cancer Treat. Res. Commun..

[261] Hoeben A., Joosten E.A.J., van den Beuken-van Everdingen M.H.J. (2021). Personalized Medicine: Recent Progress in Cancer Therapy. Cancers.

[262] Gambardella V., Tarazona N., Cejalvo J.M., Lombardi P., Huerta M., Roselló S., Fleitas T., Roda D., Cervantes A. (2020). Personalized Medicine: Recent Progress in Cancer Therapy. Cancers.

[263] Vicente A.M., Ballensiefen W., Jönsson J.-I. (2020). How Personalised Medicine Will Transform Healthcare by 2030: The ICPerMed Vision. J. Transl. Med..

[264] Saeed R.F., Awan U.A., Saeed S., Mumtaz S., Akhtar N., Aslam S. (2023). Targeted Therapy and Personalized Medicine. Therapeutic Approaches in Cancer Treatment.

[265] Anand U., Dey A., Chandel A.K.S., Sanyal R., Mishra A., Pandey D.K., De Falco V., Upadhyay A., Kandimalla R., Chaudhary A. (2023). Cancer Chemotherapy and beyond: Current Status, Drug Candidates, Associated Risks and Progress in Targeted Therapeutics. Genes Dis..

[266] Cecchin E., Stocco G. (2020). Pharmacogenomics and Personalized Medicine. Genes.

[267] Sadee W., Wang D., Hartmann K., Toland A.E. (2023). Pharmacogenomics: Driving Personalized Medicine. Pharmacol. Rev..

[268] Meric-Bernstam F., Gonzalez-Angulo A.M. (2009). Targeting the MTOR Signaling Network for Cancer Therapy. J. Clin. Oncol..

[269] Böhm R., Imseng S., Jakob R.P., Hall M.N., Maier T., Hiller S. (2021). The Dynamic Mechanism of 4E-BP1 Recognition and Phosphorylation by MTORC1. Mol. Cell.

[270] Mukhopadhyay S., Frias M.A., Chatterjee A., Yellen P., Foster D.A. (2016). The Enigma of Rapamycin Dosage. Mol. Cancer Ther..

[271] Ma L., Guo H., Zhao Y., Liu Z., Wang C., Bu J., Sun T., Wei J. (2024). Liquid Biopsy in Cancer: Current Status, Challenges and Future Prospects. Signal Transduct. Target. Ther..

[272] Sinha S., Vegesna R., Mukherjee S., Kammula A.V., Dhruba S.R., Wu W., Kerr D.L., Nair N.U., Jones M.G., Yosef N. (2024). PERCEPTION Predicts Patient Response and Resistance to Treatment Using Single-Cell Transcriptomics of Their Tumors. Nat. Cancer.

[273] Corti C., Cobanaj M., Dee E.C., Criscitiello C., Tolaney S.M., Celi L.A., Curigliano G. (2023). Artificial Intelligence in Cancer Research and Precision Medicine: Applications, Limitations and Priorities to Drive Transformation in the Delivery of Equitable and Unbiased Care. Cancer Treat. Rev..

[274] Lei Y., Tang R., Xu J., Wang W., Zhang B., Liu J., Yu X., Shi S. (2021). Applications of Single-Cell Sequencing in Cancer Research: Progress and Perspectives. J. Hematol. Oncol..

[275] Paolillo C., Londin E., Fortina P. (2019). Single-Cell Genomics. Clin. Chem..

[276] Zafar H., Wang Y., Nakhleh L., Navin N., Chen K. (2016). Monovar: Single-Nucleotide Variant Detection in Single Cells. Nat. Methods.

[277] Frumkin D., Wasserstrom A., Itzkovitz S., Harmelin A., Rechavi G., Shapiro E. (2008). Amplification of Multiple Genomic Loci from Single Cells Isolated by Laser Micro-Dissection of Tissues. BMC Biotechnol..

[278] Keays K.M., Owens G.P., Ritchie A.M., Gilden D.H., Burgoon M.P. (2005). Laser Capture Microdissection and Single-Cell RT-PCR without RNA Purification. J. Immunol. Methods.

[279] Kolodziejczyk A.A., Kim J.K., Svensson V., Marioni J.C., Teichmann S.A. (2015). The Technology and Biology of Single-Cell RNA Sequencing. Mol. Cell.

[280] Ramsköld D., Luo S., Wang Y.-C., Li R., Deng Q., Faridani O.R., Daniels G.A., Khrebtukova I., Loring J.F., Laurent L.C. (2012). Full-Length MRNA-Seq from Single-Cell Levels of RNA and Individual Circulating Tumor Cells. Nat. Biotechnol..

[281] Islam S., Kjällquist U., Moliner A., Zajac P., Fan J.-B., Lönnerberg P., Linnarsson S. (2011). Characterization of the Single-Cell Transcriptional Landscape by Highly Multiplex RNA-Seq. Genome Res..

[282] Hashimshony T., Wagner F., Sher N., Yanai I. (2012). CEL-Seq: Single-Cell RNA-Seq by Multiplexed Linear Amplification. Cell Rep..

[283] Zhao K., Li Q., Li P., Liu T., Liu X., Zhu F., Zhang L. (2024). Single-Cell Transcriptome Sequencing Provides Insight into Multiple Chemotherapy Resistance in a Patient with Refractory DLBCL: A Case Report. Front. Immunol..

[284] Chapuy B., Stewart C., Dunford A.J., Kim J., Kamburov A., Redd R.A., Lawrence M.S., Roemer M.G.M., Li A.J., Ziepert M. (2018). Molecular Subtypes of Diffuse Large B Cell Lymphoma Are Associated with Distinct Pathogenic Mechanisms and Outcomes. Nat. Med..

[285] Schmitz R., Wright G.W., Huang D.W., Johnson C.A., Phelan J.D., Wang J.Q., Roulland S., Kasbekar M., Young R.M., Shaffer A.L. (2018). Genetics and Pathogenesis of Diffuse Large B-Cell Lymphoma. N. Engl. J. Med..

[286] Diakos C.I., Charles K.A., McMillan D.C., Clarke S.J. (2014). Cancer-Related Inflammation and Treatment Effectiveness. Lancet Oncol..

[287] Roemer M.G.M., Redd R.A., Cader F.Z., Pak C.J., Abdelrahman S., Ouyang J., Sasse S., Younes A., Fanale M., Santoro A. (2018). Major Histocompatibility Complex Class II and Programmed Death Ligand 1 Expression Predict Outcome After Programmed Death 1 Blockade in Classic Hodgkin Lymphoma. J. Clin. Oncol..

[288] Liu M.-K., Liu F., Dai Y.-T., Weng X.-Q., Cheng L.-L., Fan L.-Q., Liu H., Jiang L., Sun X.-J., Fang H. (2023). Case Report: Molecular and Microenvironment Change upon Midostaurin Treatment in Mast Cell Leukemia at Single-Cell Level. Front. Immunol..

[289] Wang H., Boussouar A., Mazelin L., Tauszig-Delamasure S., Sun Y., Goldschneider D., Paradisi A., Mehlen P. (2018). The Proto-Oncogene c-Kit Inhibits Tumor Growth by Behaving as a Dependence Receptor. Mol. Cell.

[290] Ustun C., Arock M., Kluin-Nelemans H.C., Reiter A., Sperr W.R., George T., Horny H.-P., Hartmann K., Sotlar K., Damaj G. (2016). Advanced Systemic Mastocytosis: From Molecular and Genetic Progress to Clinical Practice. Haematologica.

[291] Debaize L., Jakobczyk H., Avner S., Gaudichon J., Rio A.-G., Sérandour A.A., Dorsheimer L., Chalmel F., Carroll J.S., Zörnig M. (2018). Interplay between Transcription Regulators RUNX1 and FUBP1 Activates an Enhancer of the Oncogene *c-KIT* and Amplifies Cell Proliferation. Nucleic Acids Res..

[292] Li Q., Lai Q., He C., Fang Y., Yan Q., Zhang Y., Wang X., Gu C., Wang Y., Ye L. (2019). RUNX1 Promotes Tumour Metastasis by Activating the Wnt/β-Catenin Signalling Pathway and EMT in Colorectal Cancer. J. Exp. Clin. Cancer Res..

[293] Acharya D., Mukhopadhyay A. (2024). A Comprehensive Review of Machine Learning Techniques for Multi-Omics Data Integration: Challenges and Applications in Precision Oncology. Brief. Funct. Genom..

[294] Bunnik E.M., Le Roch K.G. (2013). An Introduction to Functional Genomics and Systems Biology. Adv. Wound Care.

[295] Wu X., Yang X., Dai Y., Zhao Z., Zhu J., Guo H., Yang R. (2024). Single-Cell Sequencing to Multi-Omics: Technologies and Applications. Biomark. Res..

[296] Chakraborty S., Sharma G., Karmakar S., Banerjee S. (2024). Multi-OMICS Approaches in Cancer Biology: New Era in Cancer Therapy. Biochim. Biophys. Acta-Mol. Basis Dis..

[297] Cho S.B. (2022). Uncovering Oncogenic Mechanisms of Tumor Suppressor Genes in Breast Cancer Multi-Omics Data. Int. J. Mol. Sci..

[298] Zhao M., Zhao Z. (2016). Concordance of Copy Number Loss and Down-Regulation of Tumor Suppressor Genes: A Pan-Cancer Study. BMC Genom..

[299] Halaburkova A., Cahais V., Novoloaca A., da Silva Araujo M.G., Khoueiry R., Ghantous A., Herceg Z. (2020). Pan-Cancer Multi-Omics Analysis and Orthogonal Experimental Assessment of Epigenetic Driver Genes. Genome Res..

[300] Schupp P.G., Shelton S.J., Brody D.J., Eliscu R., Johnson B.E., Mazor T., Kelley K.W., Potts M.B., McDermott M.W., Huang E.J. (2024). Deconstructing Intratumoral Heterogeneity through Multiomic and Multiscale Analysis of Serial Sections. Cancers.

[301] Tarazona S., Arzalluz-Luque A., Conesa A. (2021). Undisclosed, Unmet and Neglected Challenges in Multi-Omics Studies. Nat. Comput. Sci..

[302] Chan Y.-T., Lu Y., Wu J., Zhang C., Tan H.-Y., Bian Z.-X., Wang N., Feng Y. (2022). CRISPR-Cas9 Library Screening Approach for Anti-Cancer Drug Discovery: Overview and Perspectives. Theranostics.

[303] Cong L., Ran F.A., Cox D., Lin S., Barretto R., Habib N., Hsu P.D., Wu X., Jiang W., Marraffini L.A. (2013). Multiplex Genome Engineering Using CRISPR/Cas Systems. Science.

[304] Asmamaw M., Zawdie B. (2021). Mechanism and Applications of CRISPR/Cas-9-Mediated Genome Editing. Biologics.

[305] Ran F.A., Hsu P.D., Lin C.-Y., Gootenberg J.S., Konermann S., Trevino A.E., Scott D.A., Inoue A., Matoba S., Zhang Y. (2013). Double Nicking by RNA-Guided CRISPR Cas9 for Enhanced Genome Editing Specificity. Cell.

[306] Vanoli F., Song E., Dermawan J.K., Fishinevich E., Sung P., Min S.S., Xie Z., de Traux de Wardin H., Hwang S., Maki R.G. (2024). Modeling Extraordinary Response Through Targeting Secondary Alterations in Fusion-Associated Sarcoma. JCO Precis. Oncol..

[307] Dermawan J.K., Vanoli F., Herviou L., Sung Y.-S., Zhang L., Singer S., Tap W.D., Benayed R., Bale T.A., Benhamida J.K. (2022). Comprehensive Genomic Profiling of EWSR1/FUS::CREB Translocation-Associated Tumors Uncovers Prognostically Significant Recurrent Genetic Alterations and Methylation-Transcriptional Correlates. Mod. Pathol..

[308] Wang S.-W., Gao C., Zheng Y.-M., Yi L., Lu J.-C., Huang X.-Y., Cai J.-B., Zhang P.-F., Cui Y.-H., Ke A.-W. (2022). Current Applications and Future Perspective of CRISPR/Cas9 Gene Editing in Cancer. Mol. Cancer.

[309] Zhang B. (2021). CRISPR/Cas Gene Therapy. J. Cell. Physiol..

[310] Xu X., Liu C., Wang Y., Koivisto O., Zhou J., Shu Y., Zhang H. (2021). Nanotechnology-Based Delivery of CRISPR/Cas9 for Cancer Treatment. Adv. Drug Deliv. Rev..

[311] Huang S., Yang J., Shen N., Xu Q., Zhao Q. (2023). Artificial Intelligence in Lung Cancer Diagnosis and Prognosis: Current Application and Future Perspective. Semin. Cancer Biol..

[312] Chen Y., Han Q., Huang Z., Lyu M., Ai Z., Liang Y., Yan H., Wang M., Xiang Z. (2022). Value of IVIM in Differential Diagnoses between Benign and Malignant Solitary Lung Nodules and Masses: A Meta-Analysis. Front. Surg..

[313] Wan Y.-L., Wu P., Huang P.-C., Tsay P.-K., Pan K.-T., Trang N., Chuang W.-Y., Wu C.-Y., Lo S. (2020). The Use of Artificial Intelligence in the Differentiation of Malignant and Benign Lung Nodules on Computed Tomograms Proven by Surgical Pathology. Cancers.

[314] Zheng B., Yang D., Zhu Y., Liu Y., Hu J., Bai C. (2021). 3D Gray Density Coding Feature for Benign-malignant Pulmonary Nodule Classification on Chest CT. Med. Phys..

[315] Mehta K., Jain A., Mangalagiri J., Menon S., Nguyen P., Chapman D.R. (2021). Lung Nodule Classification Using Biomarkers, Volumetric Radiomics, and 3D CNNs. J. Digit. Imaging.

[316] Fedorov A., Hancock M., Clunie D., Brochhausen M., Bona J., Kirby J., Freymann J., Pieper S., Aerts H.J.W.L., Kikinis R. (2020). DICOM Re-encoding of Volumetrically Annotated Lung Imaging Database Consortium (LIDC) Nodules. Med. Phys..

[317] Armato S.G., McLennan G., Bidaut L., McNitt-Gray M.F., Meyer C.R., Reeves A.P., Zhao B., Aberle D.R., Henschke C.I., Hoffman E.A. (2011). The Lung Image Database Consortium (LIDC) and Image Database Resource Initiative (IDRI): A Completed Reference Database of Lung Nodules on CT Scans. Med. Phys..

[318] Amodio V., Yaeger R., Arcella P., Cancelliere C., Lamba S., Lorenzato A., Arena S., Montone M., Mussolin B., Bian Y. (2020). EGFR Blockade Reverts Resistance to KRASG12C Inhibition in Colorectal Cancer. Cancer Discov..

[319] Coudray N., Ocampo P.S., Sakellaropoulos T., Narula N., Snuderl M., Fenyö D., Moreira A.L., Razavian N., Tsirigos A. (2018). Classification and Mutation Prediction from Non–Small Cell Lung Cancer Histopathology Images Using Deep Learning. Nat. Med..

[320] Wong E.Y., Chu T.N., Ladi-Seyedian S.-S. (2024). Genomics and Artificial Intelligence. Urol. Clin. N. Am..

[321] Liu Q., Reed M., Zhu H., Cheng Y., Almeida J., Fruhbeck G., Ribeiro R., Hu P. (2022). Epigenome-Wide DNA Methylation and Transcriptome Profiling of Localized and Locally Advanced Prostate Cancer: Uncovering New Molecular Markers. Genomics.

[322] Armakolas A., Kotsari M., Koskinas J. (2023). Liquid Biopsies, Novel Approaches and Future Directions. Cancers.

[323] Connal S., Cameron J.M., Sala A., Brennan P.M., Palmer D.S., Palmer J.D., Perlow H., Baker M.J. (2023). Liquid Biopsies: The Future of Cancer Early Detection. J. Transl. Med..

[324] Bustin S.A., Siddiqi S., Ahmed S., Hands R., Dorudi S. (2004). Quantification of Cytokeratin 20, Carcinoembryonic Antigen and Guanylyl Cyclase C MRNA Levels in Lymph Nodes May Not Predict Treatment Failure in Colorectal Cancer Patients. Int. J. Cancer.

[325] Mastoraki S., Strati A., Tzanikou E., Chimonidou M., Politaki E., Voutsina A., Psyrri A., Georgoulias V., Lianidou E. (2018). *ESR1* Methylation: A Liquid Biopsy–Based Epigenetic Assay for the Follow-up of Patients with Metastatic Breast Cancer Receiving Endocrine Treatment. Clin. Cancer Res..

[326] Newman A.M., Bratman S.V., To J., Wynne J.F., Eclov N.C.W., Modlin L.A., Liu C.L., Neal J.W., Wakelee H.A., Merritt R.E. (2014). An Ultrasensitive Method for Quantitating Circulating Tumor DNA with Broad Patient Coverage. Nat. Med..

[327] Nikanjam M., Kato S., Kurzrock R. (2022). Liquid Biopsy: Current Technology and Clinical Applications. J. Hematol. Oncol..

[328] Ma S., Zhou M., Xu Y., Gu X., Zou M., Abudushalamu G., Yao Y., Fan X., Wu G. (2023). Clinical Application and Detection Techniques of Liquid Biopsy in Gastric Cancer. Mol. Cancer.

[329] (2024). Turning the Tide of Early Cancer Detection. Nat. Med..

[330] Han H.S., Lee K.-W. (2024). Liquid Biopsy: An Emerging Diagnostic, Prognostic, and Predictive Tool in Gastric Cancer. J. Gastric Cancer.

[331] Fatima S., Ma Y., Safrachi A., Haider S., Spring K.J., Vafaee F., Scott K.F., Roberts T.L., Becker T.M., de Souza P. (2022). Harnessing Liquid Biopsies to Guide Immune Checkpoint Inhibitor Therapy. Cancers.

[332] Siravegna G., Mussolin B., Buscarino M., Corti G., Cassingena A., Crisafulli G., Ponzetti A., Cremolini C., Amatu A., Lauricella C. (2015). Erratum: Clonal Evolution and Resistance to EGFR Blockade in the Blood of Colorectal Cancer Patients. Nat. Med..

[333] Cossu A.M., Scrima M., Lombardi A., Grimaldi A., Russo M., Ottaiano A., Caraglia M., Bocchetti M. (2020). Future Directions and Management of Liquid Biopsy in Non-Small Cell Lung Cancer. Explor. Target. Anti-Tumor Ther..

[334] Canale M., Pasini L., Bronte G., Delmonte A., Cravero P., Crinò L., Ulivi P. (2019). Role of Liquid Biopsy in Oncogene-Addicted Non-Small Cell Lung Cancer. Transl. Lung Cancer Res..

[335] Pantel K., Alix-Panabières C. (2019). Liquid Biopsy and Minimal Residual Disease—Latest Advances and Implications for Cure. Nat. Rev. Clin. Oncol..

[336] Johnston A.D., Ross J.P., Ma C., Fung K.Y.C., Locke W.J. (2023). Epigenetic Liquid Biopsies for Minimal Residual Disease, What’s around the Corner?. Front. Oncol..

[337] Pantel K., Alix-Panabières C. (2025). Minimal Residual Disease as a Target for Liquid Biopsy in Patients with Solid Tumours. Nat. Rev. Clin. Oncol..

[338] Alba-Bernal A., Lavado-Valenzuela R., Domínguez-Recio M.E., Jiménez-Rodriguez B., Queipo-Ortuño M.I., Alba E., Comino-Méndez I. (2020). Challenges and Achievements of Liquid Biopsy Technologies Employed in Early Breast Cancer. eBioMedicine.

[339] Lone S.N., Nisar S., Masoodi T., Singh M., Rizwan A., Hashem S., El-Rifai W., Bedognetti D., Batra S.K., Haris M. (2022). Liquid Biopsy: A Step Closer to Transform Diagnosis, Prognosis and Future of Cancer Treatments. Mol. Cancer.

[340] Anderson N.M., Simon M.C. (2020). The Tumor Microenvironment. Curr. Biol..

[341] Saxena M., van der Burg S.H., Melief C.J.M., Bhardwaj N. (2021). Therapeutic Cancer Vaccines. Nat. Rev. Cancer.

[342] Mollica Poeta V., Massara M., Capucetti A., Bonecchi R. (2019). Chemokines and Chemokine Receptors: New Targets for Cancer Immunotherapy. Front. Immunol..

[343] Yu S., Wang S., Wang X., Xu X. (2024). The Axis of Tumor-Associated Macrophages, Extracellular Matrix Proteins, and Cancer-Associated Fibroblasts in Oncogenesis. Cancer Cell Int..

[344] Czekay R.-P., Cheon D.-J., Samarakoon R., Kutz S.M., Higgins P.J. (2022). Cancer-Associated Fibroblasts: Mechanisms of Tumor Progression and Novel Therapeutic Targets. Cancers.

[345] Kundu M., Butti R., Panda V.K., Malhotra D., Das S., Mitra T., Kapse P., Gosavi S.W., Kundu G.C. (2024). Modulation of the Tumor Microenvironment and Mechanism of Immunotherapy-Based Drug Resistance in Breast Cancer. Mol. Cancer.

[346] Kaczmarek M., Poznańska J., Fechner F., Michalska N., Paszkowska S., Napierała A., Mackiewicz A. (2023). Cancer Vaccine Therapeutics: Limitations and Effectiveness—A Literature Review. Cells.

[347] Memorial Sloan Kettering Cancer Center Cancer Vaccines: The Types, How They Work, and Which Cancers They Treat. https://www.mskcc.org/cancer-care/diagnosis-treatment/cancer-treatments/immunotherapy/cancer-vaccines.

[348] Liu J., Fu M., Wang M., Wan D., Wei Y., Wei X. (2022). Cancer Vaccines as Promising Immuno-Therapeutics: Platforms and Current Progress. J. Hematol. Oncol..

[349] Miao L., Zhang Y., Huang L. (2021). MRNA Vaccine for Cancer Immunotherapy. Mol. Cancer.

[350] Pérez-Baños A., Gleisner M.A., Flores I., Pereda C., Navarrete M., Araya J.P., Navarro G., Quezada-Monrás C., Tittarelli A., Salazar-Onfray F. (2023). Whole Tumour Cell-Based Vaccines: Tuning the Instruments to Orchestrate an Optimal Antitumour Immune Response. Br. J. Cancer.

[351] Pandya A., Shah Y., Kothari N., Postwala H., Shah A., Parekh P., Chorawala M.R. (2023). The Future of Cancer Immunotherapy: DNA Vaccines Leading the Way. Med. Oncol..

[352] Rastogi I., Muralidhar A., McNeel D.G. (2023). Vaccines as Treatments for Prostate Cancer. Nat. Rev. Urol..

[353] Madan R.A., Gulley J.L. (2011). Sipuleucel-T: Harbinger of a New Age of Therapeutics for Prostate Cancer. Expert Rev. Vaccines.

[354] Han J., Gu X., Li Y., Wu Q. (2020). Mechanisms of BCG in the Treatment of Bladder Cancer-Current Understanding and the Prospect. Biomed. Pharmacother..

[355] Jiang S., Redelman-Sidi G. (2022). BCG in Bladder Cancer Immunotherapy. Cancers.

[356] Cheng L., Wang Y., Du J. (2020). Human Papillomavirus Vaccines: An Updated Review. Vaccines.

[357] Aggarwal S., Agarwal P., Singh A.K. (2023). Human Papilloma Virus Vaccines: A Comprehensive Narrative Review. Cancer Treat. Res. Commun..

[358] Flores J.E., Thompson A.J., Ryan M., Howell J. (2022). The Global Impact of Hepatitis B Vaccination on Hepatocellular Carcinoma. Vaccines.

[359] Cao M., Fan J., Lu L., Fan C., Wang Y., Chen T., Zhang S., Yu Y., Xia C., Lu J. (2022). Long Term Outcome of Prevention of Liver Cancer by Hepatitis B Vaccine: Results from an RCT with 37 Years. Cancer Lett..

[360] Kaufman H.L., Kohlhapp F.J., Zloza A. (2015). Oncolytic Viruses: A New Class of Immunotherapy Drugs. Nat. Rev. Drug Discov..

[361] Thorne S.H., Liang W., Sampath P., Schmidt T., Sikorski R., Beilhack A., Contag C.H. (2010). Targeting Localized Immune Suppression Within the Tumor Through Repeat Cycles of Immune Cell-Oncolytic Virus Combination Therapy. Mol. Ther..

[362] Ma J., Ramachandran M., Jin C., Quijano-Rubio C., Martikainen M., Yu D., Essand M. (2020). Characterization of Virus-Mediated Immunogenic Cancer Cell Death and the Consequences for Oncolytic Virus-Based Immunotherapy of Cancer. Cell Death Dis..

[363] Guo Z.S., Liu Z., Bartlett D.L. (2014). Oncolytic Immunotherapy: Dying the Right Way Is a Key to Eliciting Potent Antitumor Immunity. Front. Oncol..

[364] Yan Z., Zhang Z., Chen Y., Xu J., Wang J., Wang Z. (2024). Enhancing Cancer Therapy: The Integration of Oncolytic Virus Therapy with Diverse Treatments. Cancer Cell Int..

[365] Zhu Z., McGray A.J.R., Jiang W., Lu B., Kalinski P., Guo Z.S. (2022). Improving Cancer Immunotherapy by Rationally Combining Oncolytic Virus with Modulators Targeting Key Signaling Pathways. Mol. Cancer.

[366] Raja J., Ludwig J.M., Gettinger S.N., Schalper K.A., Kim H.S. (2018). Oncolytic Virus Immunotherapy: Future Prospects for Oncology. J. Immunother. Cancer.

[367] Ferrucci P.F., Pala L., Conforti F., Cocorocchio E. (2021). Talimogene Laherparepvec (T-VEC): An Intralesional Cancer Immunotherapy for Advanced Melanoma. Cancers.

[368] Peter M., Kühnel F. (2020). Oncolytic Adenovirus in Cancer Immunotherapy. Cancers.

[369] Hemminki O., dos Santos J.M., Hemminki A. (2020). Oncolytic Viruses for Cancer Immunotherapy. J. Hematol. Oncol..

[370] Russell S.J., Barber G.N. (2018). Oncolytic Viruses as Antigen-Agnostic Cancer Vaccines. Cancer Cell.

[371] Fan T., Zhang M., Yang J., Zhu Z., Cao W., Dong C. (2023). Therapeutic Cancer Vaccines: Advancements, Challenges and Prospects. Signal Transduct. Target. Ther..

[372] Zheng M., Huang J., Tong A., Yang H. (2019). Oncolytic Viruses for Cancer Therapy: Barriers and Recent Advances. Mol. Ther.-Oncolytics.

